# Globular Adiponectin Limits Microglia Pro-Inflammatory Phenotype through an AdipoR1/NF-κB Signaling Pathway

**DOI:** 10.3389/fncel.2017.00352

**Published:** 2017-11-14

**Authors:** Sarah Nicolas, Julie Cazareth, Hadi Zarif, Alice Guyon, Catherine Heurteaux, Joëlle Chabry, Agnès Petit-Paitel

**Affiliations:** Centre Nationnal de la Recherche Scientifique, UMR7275 Institut de Pharmacologie Moléculaire et Cellulaire, Université Côte d'Azur, Valbonne, France

**Keywords:** microglia, astrocytes, brain, neuroinflammation, adipokines, cytokines, nitrosative stress, oxidative stress

## Abstract

We recently reported that increased levels of Adiponectin (ApN) in the brain led to microglia phenotype and activation state regulation, thus reducing both global brain inflammation and depressive-like behaviors in mice. Apart from this, little is known on ApN molecular effects on microglia, although these cells are crucial in both physiological and pathological processes. Here we fill this gap by studying the effects and targets of ApN toward neuroinflammation. Our findings suggest that ApN deficiency in mice leads to a higher sensitivity of mice to neuroinflammation that is due to enhanced microglia responsiveness to a pro-inflammatory challenge. Moreover, we show that globular ApN (gApN) exerts direct *in vivo* anti-inflammatory actions on microglia by reducing IL-1β, IL-6, and TNFα synthesis. *In vitro*, gApN anti-inflammatory properties are confirmed in brain-sorted microglia, primary cultured and microglia cell line (BV2), but are not observed on astrocytes. Our results also show that gApN blocks LPS-induced nitrosative and oxidative stress in microglia. Finally, we demonstrate for the first time that these anti-inflammatory and anti-oxidant actions of gApN on microglia are mediated through an AdipoR1/NF-κB signaling pathway.

## Introduction

Chronic neuroinflammation in the brain is a self-sustaining and long-lasting neuroinflammatory response characterized not only by a continuous activation of microglia, astrogliosis, and continuous release of inflammatory mediators, but also by increased oxidative and nitrosative stress (Block and Hong, 2005). This leads to blood-brain barrier (BBB) damage, resulting in infiltration of peripheral monocytes into the brain what maintains or even amplifies the inflammation (Rivest, 2009). Chronic neuroinflammation is most often harmful to neurons. Neurodegenerative diseases such as multiple sclerosis (MS), Alzheimer's disease (AD), Parkinson's disease (PD), and Huntington's disease (HD), but also psychiatric disorders, including major depressive disorders (MDD) are associated with chronic neuroinflammation (Dantzer et al., 2008; Frank-Cannon et al., 2009).

Chronic neuroinflammation may be the consequence of a chronic low-grade inflammation status in peripheral organs. Lifestyle factors, such as physical inactivity, smoking habits, obesity, insulin resistance, non-healthy diets, and vitamin D deficiency may affect the immune system and lead to the establishment of inflammation. Conversely, favorable living conditions that provide a set of cognitive, social, sensory stimulations with physical exercise can help fighting against chronic inflammation. In periphery, a molecule known for its anti-inflammatory properties is Adiponectin (ApN). ApN is the most abundant adipokine (Arita et al., 1999; Matsuzawa, 2005), almost exclusively secreted by adipocytes (Scherer et al., 1995; Maeda et al., 1996). At the peripheral level, ApN is involved in many physiological processes, including the regulation of energy metabolism, vascular physiology, and inflammation (Kadowaki and Yamauchi, 2005). Its effects are considered to be anti-atherogenic and anti-diabetogenic (Gil-Campos et al., 2004). The plasma ApN rates are inversely correlated with the amount of visceral adipose tissue (Weyer et al., 2001; Kern et al., 2003). Cardiometabolic diseases such as metabolic syndrome, atherosclerosis and type 2 diabetes are associated with a low-grade chronic inflammatory condition, as well as low levels of circulating ApN (Hotta et al., 2000; Hoffstedt et al., 2004; Hara et al., 2006). The generally accepted hypothesis is that ApN would be an anti-inflammatory molecule whose low plasma levels would contribute to the establishment of a chronic inflammatory condition (Maeda et al., 2002; Xu et al., 2003).

In periphery, the main cellular targets of ApN are hepatocytes, cardiac myocytes, pancreatic beta cells, macrophages/monocytes, and podocytes. At the central level, there is much less data available. Although this has been subject to debate, it is now accepted that ApN crosses the BBB and reaches the brain parenchyma especially in the hypothalamus (Kubota et al., 2007; Guillod-Maximin et al., 2009; Nicolas et al., 2015) and hippocampus (Jeon et al., 2009; Yau et al., 2014) where it modifies the activation of neurons (Kubota et al., 2007; Zhang et al., 2011, 2016a,b; Liu et al., 2012; Yau et al., 2014; Nicolas et al., 2015), microglia (Chabry et al., 2015) and astrocytes (Wan et al., 2014).

ApN is a 30-kDa protein that forms multimeric complexes that combine via its collagen domain to create three main circulating isoforms: a trimer (low molecular weight LMW), a hexamer of medium molecular weight (MMW) and a larger multimeric high molecular weight (HMW) form (Pajvani and Scherer, 2003; Tsao et al., 2003). A proteolytic cleavage product of ApN, known as globular ApN (gApN), also circulates at very low levels in plasma (Fruebis et al., 2001; Waki et al., 2005). The functional role of each of these isoforms is not formally known, although the HMW ApN is considered as the main determinant of insulin sensitivity and the LMW ApN is the only form found in the cerebrospinal fluid (CSF) (Ebinuma et al., 2007; Kusminski et al., 2007; Chabry et al., 2015).

ApN exerts multiple physiological effects by binding to its receptors, AdipoR1, AdipoR2 (Yamauchi et al., 2003, 2007; Kadowaki and Yamauchi, 2005). AdipoR1 and AdipoR2 play important roles in the regulation of inflammation, glucose and lipid metabolism, and oxidative stress (Yamauchi et al., 2003, 2007, 2014; Chinetti et al., 2004; Yamaguchi et al., 2005). Both are present in peripheral monocytes/macrophages with high abundance of AdipoR1 (Chinetti et al., 2004; Yamaguchi et al., 2005; Hui et al., 2015), however their precise respective contribution to the anti-inflammatory action of ApN remains unclear. Indeed, AdipoR1 predominantly binds to gApN and mediates the suppression of Nuclear Factor-κB (NF-κB) activation and the subsequent reduction of expression of pro-inflammatory cytokines (Yamaguchi et al., 2005; Mandal et al., 2011) whereas AdipoR2 mediates the M2 anti-inflammatory phenotype of macrophages induced by ApN (Mandal et al., 2011).

Although less documented than its peripheral functions, ApN also has essential role in the CNS. Interestingly, we and others recently described ApN as being a major contributor to the antidepressant effects of enriched environment (EE), an experimental paradigm for laboratory animals that mimics stimulating and positive living conditions, characterized by social interactions, cognitive, sensory, and motor stimuli and voluntary exercise (Yau et al., 2014; Nicolas et al., 2015). We demonstrated that in the mouse brain, EE-mediated ApN level increase influenced microglial activation state to an anti-inflammatory phenotype, which significantly and concomitantly reduced the neuroinflammation and anxiety/depressive-like behaviors (Chabry et al., 2015).

Although anti-inflammatory effects of ApN on blood-monocytes and macrophages are known, no information is available yet on the direct influence of ApN on microglial activation. To fill this gap, we conducted this study which demonstrates that gApN has powerful anti-inflammatory, anti-nitrosative and anti-oxidant properties on LPS-stimulated microglia through an AdipoR1-NF-κB signaling pathway.

## Materials and methods

### Animals

Six weeks-old male wt, adiponectin knock-out (ApN^−/−^) or adiponectin-receptor 2 knock-out (AdipoR2^−/−^) mice with the same C57BL/6J genetic background were housed at 22°C with a 12-h light-dark cycle (lights on at 07:00) with free access to drink and chow (A04, SAFE). All experiments were conducted in compliance with the Institutional Animal Care and Use Committee of the University of Nice-Sophia Antipolis (permission number 010344.01 from the French “Ministère de l'Enseignement Supérieur et de la Recherche”).

### LPS administration

Lipopolysaccharide (LPS) from *Escherichia coli* 0111:B4 was purchased from Sigma-Aldrich and freshly dissolved in sterile saline prior to i.p. injection at a dose of 1 mg/kg.

### I.c.v injections of APN

Wt mice were anesthetized with a mixture of ketamine (100 mg/kg) and xylazine (10 mg/kg). The mouse was placed on a stereotaxic apparatus (Kopf) on heating mat and the skull was exposed. I.c.v injections (0.3 μg of ApN in 2 μl of NaCl 0.9% or vehicle alone) were performed using a Hamilton micro-syringe (cerebral lateral ventricle coordinates: −0.22 mm posterior and 1 mm lateral to the bregma; depth 2.0 mm) at 0.2 μl/min.

### CSF sampling and cytokine measurement

Mice were anesthetized with an i.p. injection of sodium pentobarbital; then CSF (2–5 μl) was obtained by cisternal puncture. Multiplex measurement of proinflammatory cytokines in mouse CSF was performed with the MSD 96-well multi-array mouse cytokine assay (Mesoscale). For comparison, data were normalized relative to the CSF volume.

### Isolation of immune cells from adult mouse brains

Mice were deeply anesthetized with a lethal injection of pentobarbital. Immune brain cells were isolated from whole-brain homogenates using a protocol adapted from Cardona et al. (2006), as previously described in Cazareth et al. (2014). Mice were transcardially perfused with ice-cold HBSS containing 1 mg/ml EDTA. Brains were collected and roughly homogenized in PBS, resuspended in PBS containing 3 mg/ml collagenase D (Roche Diagnostics) and incubated 20 min at 37°C. After incubation, brain homogenates were filtered on 70 μm pore size cell strainers (BD Biosciences), centrifuged (10 min, 2,000 rpm), washed and resuspended in 6 ml of 38% isotonic Percoll (GE Healthcare), before centrifugation (20 min, 2,000 rpm, 4°C). The surface ring containing myelin and debris was discarded. Cell pellets containing brain immune cells were collected, washed with PBS containing 0.5% BSA and 2.5 mM EDTA and labeled for subsequent cell sorting and/or flow cytometry analysis.

### Brain immune cell staining, flow cytometry, and cell sorting

Staining of brain immune cell surface antigens was performed as previously described (Cazareth et al., 2014). Briefly, Fc receptors were blocked with 2.4G2 antibody. Microglia was identified according to the labeling of CD11b-PercP-Cy5.5 and CD45-APC-Cy7 conjugated antibodies (BD Biosciences). Inflammatory monocytes were defined as CD11b^+^CD45^hi^Ly-6G^neg^Ly-6C^hi^ cells (Ly-6G and Ly-6C antibodies were purchased from BD Biosciences). Cells were washed and resuspended in PBS containing 0.5% BSA and 2.5 mM EDTA for analysis and cell sorting with FACS Aria III (BD Biosciences).

### *Ex vivo* culture of microglia

Brain-sorted microglia was seeded at a density of 2 × 10^4^ cells/well in 96-well tissue culture plates (Falcon) in Dulbecco's Modified Eagle's Medium (DMEM) culture media (Gibco) containing 10% fetal bovine serum (FBS, Gibco). Cells were cultured at 37°C with 5% CO_2_ and saturated humidity for 24 h. Fifteen minutes after cell plating, gApN (10 μg/ml, Phoenix Pharmaceuticals) was added, 1 h prior stimulation with LPS (0.5 μg/ml). LPS from *Escherichia coli* 0111:B4 was purchased from Sigma-Aldrich and freshly dissolved in sterile saline prior to cell treatment. Media were then collected and centrifuged at 1,200 rpm for 10 min at 4°C.

### Tissue collection and immunohistochemistry

Mice were deeply anesthetized with pentobarbital and transcardially perfused with PBS and then 3.2% paraformaldehyde. Brains were postfixed in 3.2% paraformaldehyde for 48 h. Fixed brains were sectioned (40 μm) using a vibratome (Leica). Sections were blocked with 3% goat serum. Sections were washed and incubated with a rabbit anti-mouse ionized calcium binding adaptor molecule 1 (Iba-1) antibody (Wako Chemicals, USA), overnight at 4°C, followed by incubation with the appropriate fluorescent-conjugated secondary antibody (Invitrogen). Slices were then mounted on glass slices with Fluoroprep (Dako) containing 1 mg/ml Hoescht, a fluorescent specific DNA dye (Invitrogen). Each fluorochrome was independently captured with an FV10i scanning confocal microscope (Olympus, France). Quantification of Iba-1 staining was performed using ImageJ software (NIH). The software generated fluorescence intensity values by tracing the region-of-interest (ROI). Arbitrary units were defined in terms of strength of fluorescent signal. The final intensity values were calculated by subtracting the area of the selected region multiplied by the background fluorescence from the fluorescence intensity of the region-of-interest (ROI): fluorescence intensity (arbitrary units) = fluorescence intensity of ROI − (area of selected region × mean fluorescence of background).

### Primary astrocytes and microglia cell cultures

Primary microglial cell cultures were prepared from the brains of newborn C56BL/6J mice (postnatal days 1–2; Marella and Chabry, 2004). The brains were removed and placed in DMEM, then the meninges and blood vessels were removed under a microscope. After mechanical dissociation, aliquots of the cell suspension were plated in a poly-D-lysine coated flask (Falcon). Cultures containing mainly astrocytes (90%) and microglial cells (10%) were maintained at 37°C in a humidified 5% CO2 atmosphere for 10 days, with the change of the medium every 3–4 days. The cultures were then shaken on an orbital shaker at 150 rpm for 60 min at 37°C. Floating cells in the supernatant were collected, centrifuged at 250 g for 5 min, and resuspended in the culture medium. This fraction contained the microglial cells (around 93% of purity). The adherent cell population (i.e., astrocytes) was grown in DMEM containing 10% FCS, split every week and used after two passages. Astrocytes cultures contained around 99% of GFAP+ positive cells.

### Cell culture

BV2 mouse microglia cells were grown and maintained in DMEM (4.5 g/l glucose, 4 mM L-glutamine, without sodium pyruvate from Gibco) supplemented with 10% fetal bovine serum (FBS, Gibco), 100 U/ml penicillin and 100 μg/ml streptomycin (Gibco). Cells were grown in an atmosphere of 5% CO2 at a temperature of 37°C. AdipoRon (2-(4-benzoylphenoxy)-N-[1-(phenylmethyl)-4 piperidinyl]acetamide) was purchased from Sigma-Aldrich and dissolved in 2.5% DMSO.

### siRNA cell transfection

AdipoR1 or AdipoR2 interfering RNA (siRNA) or scrambled siRNA (Qiagen) were transfected into BV2 cells using RNAiMax Lipofectamine (Invitrogen) according to the manufacturer's recommendations. After various experimental tests, 48 h post-transfection time was selected as the optimal duration to allow maximal decrease of targeted protein expression. Therefore, in all experiments, cells were used 48 h after siRNA transfection.

### Immunocytochemistry

BV2 cells were grown on poly-D-lysine-coated glass coverslips. After treatment, cells were fixed with 3.2% paraformaldehyde, then permeabilized in PBS pH 7.4, containing 0.3% Tween and 3% BSA. Cells were then incubated overnight with the appropriate primary antibody directed against CD11b (Abcam), iNOS (Merck Millipore), AdipoR1 or AdipoR2 (Sigma Aldrich) in the same buffer. At the end of the incubation time, cells were washed three times with PBS and incubated with either Alexa488-conjugated or Alexa594-conjugated secondary antibodies (Invitrogen) and Hoechst fluorescent stain (Invitrogen) for 1 h at room temperature. For NF-κB p65 subunit nuclear translocation study, cells were pre-treated with 10 μg/ml gApN or saline for 1 h before addition of 0.5 μg/ml LPS for 3 additional hours. Cells were then fixed and permeabilized as previously. Primary antibody against p65 was from Cell Signaling. Images were captured with an FV10i scanning confocal microscope (Olympus, France) and analyzed using Image J software (“National Institutes of Health” Image J Software™).

### RNA isolation and quantitative PCR

Total RNA from hippocampus, hypothalamus, amydgala, prefrontal cortex, microglia or astrocytes were isolated using the Trizol® RNA extraction kit (Invitrogen) according to the manufacturer recommendations followed by a RQ1 DNAse (Promega) treatment. First-strand cDNA were synthesized from 2 μg of total RNA with 200U of SuperScript III reverse transcriptase (SuperScriptIII, Invitrogen) in the appropriate buffer in the presence of 25 μg/ml random primers, 0.5 mM desoxyribonucleotide triphosphate mix, 5 mM dithiothreitol, 40U RNAsin (Promega). The reaction was incubated 5 min at 25°C, then 50 min at 50°C then inactivated 15 min at 70°C. Quantitative PCR was performed using the SYBRgreen method (Roche) with the LightCycler 480 sequence detector system (Roche Diagnostics). β-actin and GAPDH were used as reference genes for normalization. Primers were purchased from QIAGEN (QuantiTect primer assay, QIAGEN).

### *Ex vivo* supernatants collection and cytokine measurement by CBA or MSD

Supernatants from *ex vivo* microglia or BV2 cells were harvested and the concentration of secreted cytokines (TNFα, IL-1β, and IL-6) was detected using a cytometric bead array according to the manufacturer's instructions (BD Biosciences) or with the MSD 96-well multi-array mouse cytokine assay (Mesoscale), as specified for each set of experiments. For comparison, data were normalized relative to the cell number.

### Measurement of intracellular ROS (reactive oxygen species)

BV2 cells were seeded in 24-well plates as described above and then pre-treated with 10 μg/ml gApN for 1 h, then LPS (0.5 μg/ml) was added. Fifteen hours later, 2.7-dichlorofluorescin diacetate (DCFH-DA) (10 μM; Sigma-Aldrich) was added and incubated at 37°C, 5% CO_2_. DCFH-DA is a redox-sensitive dye which is readily taken up by the cells. Within the cell, esterases cleave the acetate groups on DCFH-DA, thus trapping the reduced form of the probe (DCFH), intracellularly. ROS in the cells oxidize DCFH, yielding the fluorescent product DCF. After 60 min, culture media were removed and kept at 4°C for subsequent NO production assay. Cells were rinsed with PBS and harvested in Trypsin/EDTA (Gibco). Cells were pelleted and resuspended in PBS containing 0.5% BSA and 2.5 mM EDTA for FACS analysis (Fortessa BD Biosciences).

### Measurement of nitric oxide production

NO2^−^ was measured in BV2 culture supernatants to assess the NO production in microglial cells. Fifty microliters of the culture media were mixed with 50 μl of Griess' reagent for nitrite from Promega in a 96-well plate and incubated at 25°C for 10 min. The absorbance at 570 nm was measured by a microplate reader. Sodium nitrite (NaNO2) was used as the standard to calculate the NO2^−^ concentrations.

### Cell homogenates, subcellular fractionation, and western blotting

For total protein extraction, BV2 cells were rinsed with PBS and homogenized in lysis buffer (50 mM Tris-HCl, pH 7.5, 150 mM NaCl, 5 mM EDTA containing 0.5% Triton X-100 and 0.5% sodium deoxycholate). Equal amounts of total proteins (40 μg) determined by the Bradford method (BioRad) were separated onto 12% SDS-PAGE, then transferred on nitrocellulose membrane (Schleicher & Schuell, Dassel, Germany). Blots were incubated in PBS 0.1% Tween, 1% milk with antibodies directed against phospho-AMPKα (Thr172), total AMPK (Cell Signaling), iNOS/NOSII (Merck Millipore), or COX-2 (Chemicon), and then with the appropriate HRP-secondary antibody. Blots were developed using an enhanced chemoluminescence system (Immobilon, Merck Millipore) with a Fusion detector (Vilber). To correct for any loading artifact, same blots were re-probed with anti-actin antibody (Abcam). Densitometry analyses were performed with a “National Institutes of Health” Image J Software™ on the immuno-positive bands. Results were expressed as a percentage of control load. For NF-κB translocation study, cells were pre-treated for 1 h with 10 μg/ml gApN or saline before addition of 0.5 μg/ml LPS for 3 additional hours. Cells were then rinsed with PBS and detached with trypsin/EDTA (Gibco). After centrifugation, cells were homogenized in lysis buffer [10 mM Hepes, pH 7.4, 10 mM KCl, 0.1 mM EDTA, 0.5% NP-40, 1 mM dithiothreitol (DTT), and protease inhibitor cocktail from Roche]. Cells were incubated 20 min on ice and then centrifuged for 10 min at 12,000 g. The supernatants contained cytosolic proteins. For nuclear protein extraction, nuclear pellets were homogenized in extraction buffer (20 mM Hepes pH 7.4, 400 mM NaCl, 1 mM EDTA, 1 mM DTT, and protease inhibitor cocktail). After 30 min on ice, samples were centrifuged 15 min at 12,000 g, 4°C. Supernatants contained nuclear extracts. Equal amounts of protein of nuclear extracts were loaded on 10% SDS-PAGE then transferred to nitrocellulose as previously described. Blots were probed with antibody against NF-κB p65 subunit from Cell Signaling. Histone H3 immunoreactivity (Cell Signaling) was used as a nuclear loading control.

### Statistical analysis

Statistical analysis was performed using the XLSTAT software. Significant differences between two groups of data were determined using a Mann & Whitney test for non-parametric data. Alternatively, Kruskal–Wallis analysis followed by a Conover-Iman test for multiple comparisons with Bonferroni correction between two independent groups, or a two-way ANOVA was applied. Results from data analysis were expressed as mean ± standard error of the mean (SEM). Statistical significance was set at ^*^P < 0.05 and ^**^P < 0.01, ^***^P < 0.001.

## Results

### ApN-deficient mice are more sensitive to LPS-induced neuroinflammation than Wt mice

**Figure 1:**
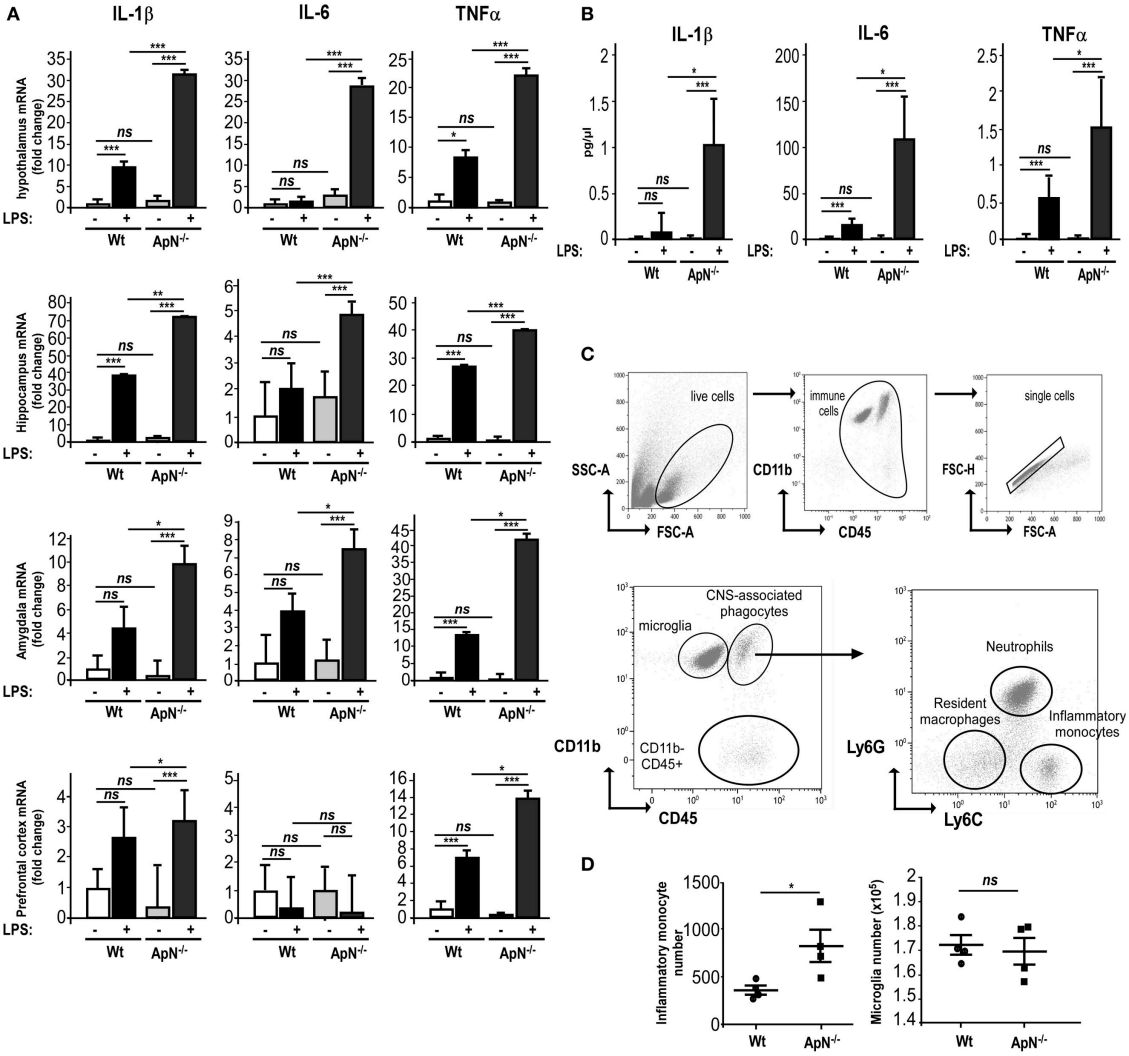
Adiponectin deficiency exacerbates neuroinflammatory responses to LPS ip administration. **(A)** Twenty-four hours post saline or LPS ip administration, brains from Wt (white and gray bars, respectively) or ApN^−/−^ mice (black and dark gray bars, respectively) were dissected and pro-inflammatory genes IL-1β, IL-6, and TNFα mRNA levels were determined by quantitative PCR from hypothalamus, hippocampus, amygdala, and prefrontal cortex. Bars represent the mean expression levels ± s (where s = √ (s1^2^+ s2^2^), s1 = standard deviation of the ΔCt of the gene of interest among replicates and s2 = standard deviation of the Ct of the housekeeping genes among replicates) expressed as fold change compared to saline injected Wt mouse brains. Kruskal–Wallis test followed by the Iman-Conover method for multiple comparison between groups was performed on ΔCt values, ^*^p < 0.05, ^**^p < 0.01, ^***^p < 0.005 vs. saline control; ns, non-significant, *n* = 4–5. **(B)** Concentrations of IL-1β, IL-6, and TNFα cytokines in CSF of saline and LPS-treated Wt mice (white and black bars, respectively) or saline and LPS-treated ApN^−/−^ mice (light gray and dark gray bars, respectively) were measured as indicated in the Materials and Methods section. *N* = 4 *per* group, ns, non-significant; Mann & Whitney for comparison between groups, ^*^p < 0.05, ^***^p < 0.005. **(C)** Representative bivariate dot plots of Percoll isolated brain cells illustrating gating strategy on CD11b^+^/CD45^low+^ for microglia, CD11b^+^/CD45^high+^ for CNS-associated phagocytes and CD11b^−^/CD45^high+^ cells. Among CNS-associated phagocytes, three subpopulations can be discriminated, i.e., CD11b^+^/CD45^high+^/Ly6G^−^/Ly6C^−^ resident monocytes, CD11b^+^/CD45^high+^/Ly6G^+^/Ly6C^med^ neutrophils and CD11b^+^/CD45^high+^/Ly6G^−^/Ly6C^+^ inflammatory monocytes. **(D)** Immune brain cells were isolated and analyzed as described in the Materials and Methods section. Dot plots show the number of inflammatory monocytes (left panel) and microglia (right panel) in Wt and ApN^−/−^ mouse brains after LPS challenge. Bars represent mean ± SEM, *n* = 4 *per* group.

In order to explore how ApN influences brain inflammation, we first evaluated the impact of ApN deficiency on mouse brain susceptibility to neuroinflammation. We analyzed the effect of a single LPS i.p. administration on pro-inflammatory cytokine mRNA expression in different brain regions of wild-type (Wt) and ApN^−/−^ mice. Brain regions were selected according to their implication in emotional stress information (amydgala, hippocampus, prefrontal cortex) or integration (hypothalamus). While IL-6 mRNA levels were not modified 24 h after LPS treatment in Wt mice regardless the brain area, IL-1β and TNFα mRNA levels were elevated in all the analyzed brain regions of Wt and ApN^−/−^ mice, although to different extents depending on the area (Figure 1A). After the LPS challenge, IL-1β, IL-6, and TNFα mRNA levels were statistically higher in all brain regions from ApN^−/−^ mice than in those from Wt mice, except from IL-6 mRNAs in prefrontal cortex (Figure 1A). We also investigated the levels of pro-inflammatory cytokines IL-1β, IL-6, and TNFα in the CSF of Wt and ApN^−/−^ mice, 24 h after saline or LPS ip administration (Figure 1B). As expected, pro-inflammatory cytokines levels were extremely low in NaCl-injected Wt and ApN^−/−^ mouse CSF samples. LPS induced a strong increase of IL-1β, IL-6, and TNFα levels in the CSF of Wt and ApN^−/−^ mice that was statistically higher in ApN^−/−^ mice as compared to Wt mice. Anti-inflammatory cytokine IL-10 was also detectable in the CSF of all mouse groups, but its levels were similarly increased after LPS challenge in Wt and ApN^−/−^ CSF samples (data not shown).

Microglia is defined as being CD11b^+^/CD45^low+^ while the heterogeneous population of CNS-associated phagocytes is CD11b^+^/CD45^high+^. The gating strategy for their correct identification is presented in Figure 1C. Three different populations were identified in the brain immune cell suspensions: CD11b^+^/CD45^high+^ (CNS-associated phagocytes), CD11b^+^/CD45^low+^ (microglia), and CD11b^−^/CD45 ^high+^ cells (Figure 1C).

CNS infiltration of CD11b^+^CD45^high+^Ly-6C^hi^ monocytes is a hallmark of CNS inflammation, including that caused by a peripheral injection of LPS. Flow cytometry analysis revealed that 24 h post LPS injection, the number of CD11b^+^CD45^high+^Ly-6C^hi^ inflammatory monocytes was statistically higher in ApN^−/−^ mice as compared to Wt mice. The number of microglia collected from whole brains was similar in both mouse groups (Figure 1D).

Altogether, these results indicated that ApN deficiency triggers a stronger global neuroinflammatory response after a peripheral LPS challenge as compared to Wt mice, suggesting anti-inflammatory properties of ApN within the CNS.

### Microglia from ApN-deficient mice is more sensitive to LPS-induced inflammation than microglia from Wt mice

**Figure 2:**
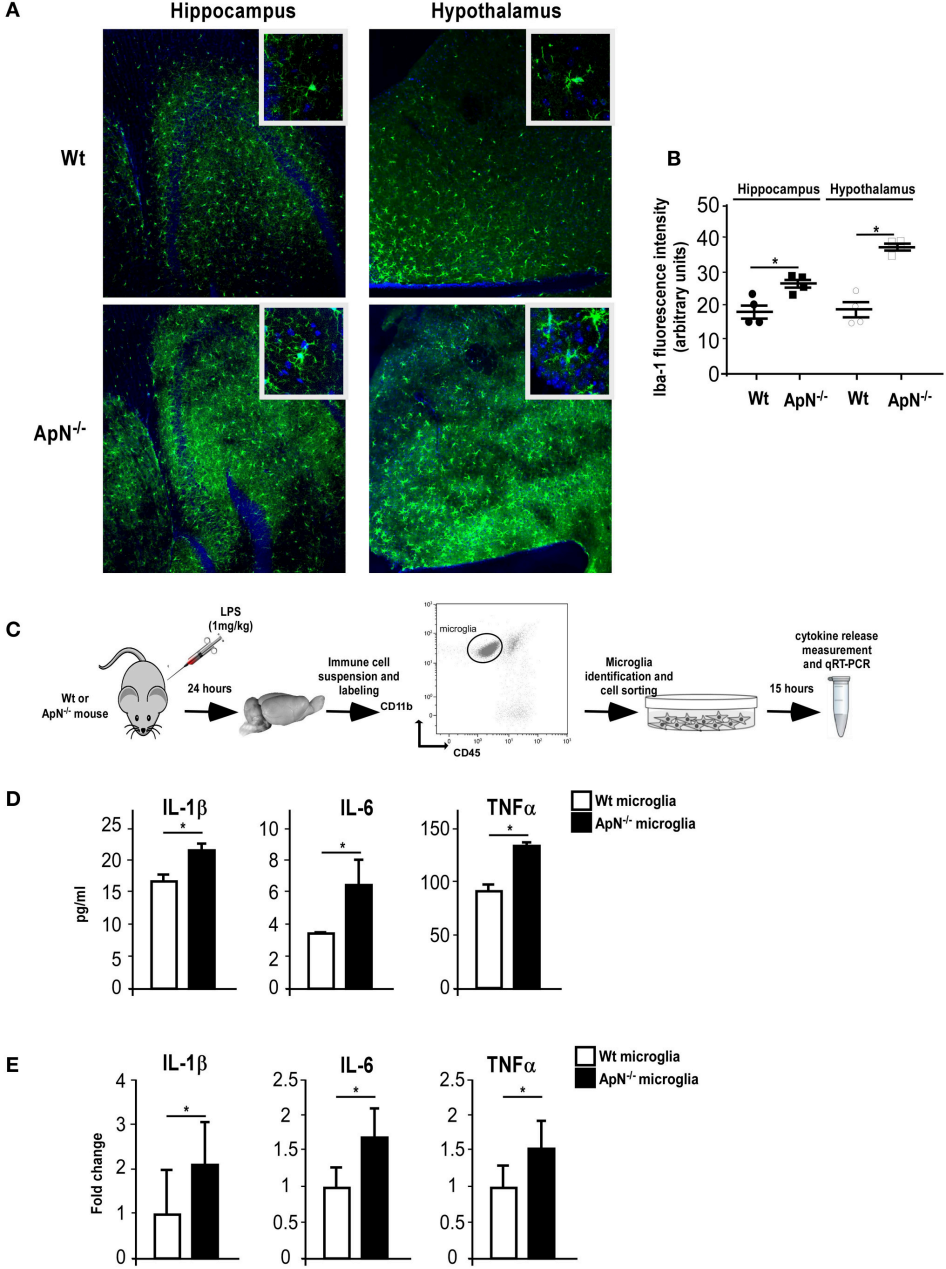
Adiponectin deficiency leads to microglia hyper-responsiveness toward LPS. **(A)** Representative images of Iba-1-immunoreactive microglia (green fluorescence) in hippocampus (left panels) and hypothalamus (right panels) from Wt (upper panels) and ApN^−/−^ (lower panels) mouse brains, 24 h after LPS challenge. Nuclei are stained with Hoechst fluorescent dye (blue fluorescence). All images were taken under the same parameters. **(B)** Quantification of Iba-1 staining using fluorescence intensity values to quantify Iba-1 levels. **(C)** Schematic representation of the protocol. 24 h after LPS ip injection, IL-1β, IL-6, and TNFα proteins **(D)** or mRNAs **(E)** were quantified by CBA or qRT-PCR, respectively, from microglia sorted from brain cell suspensions of Wt (white bars) or ApN^−/−^ mice (black bars) cultured for 15 h. **(D)** Data represent the mean concentration ± SEM, *n* = 4. **(E)** Data represent the mean expression levels ± s expressed as fold change compared to LPS-treated Wt mouse microglia, *n* = 4. Mann & Whitney for comparison between groups, ^*^p < 0.05, vs. control.

Microglia, the immune-like cells of the brain, plays an important role in inflammatory responses in the CNS. Previously, we have shown that EE mediates anti-inflammatory effects in the brain by targeting microglia activation profile in an ApN-dependent manner (Chabry et al., 2015). Thus, we investigated whether the greater susceptibility of ApN^−/−^ mice toward neuroinflammation may be due to a different responsiveness of microglia caused by ApN deficiency. To answer this question, we subjected Wt and ApN^−/−^ mice to a single LPS i.p. injection (1 mg/kg) and analyzed microglia reactivity by immunohistochemistry, protein analysis and qRT-PCR. Figure 2A shows Iba-1 labeling of microglia in hippocampus and hypothalamus of Wt and ApN^−/−^ mouse brains, 24 h after LPS ip administration. Quantification of images shows that in both brain regions, Iba-1 fluorescence appeared more intense in ApN^−/−^ challenged mice than in Wt mice (Figure 2B).

Microglia was sorted out of LPS-challenged brains from Wt and ApN^−/−^ mice (Figure 2C). Protein analysis of the secretion media of brain-sorted microglia revealed that LPS-stimulated microglia sorted from ApN^−/−^ mice produced statistically more IL-1β, IL-6, and TNF-α pro-inflammatory cytokines than that from Wt mice (Figure 2D). Similarly, qRT-PCR analysis of pro-inflammatory cytokine mRNA expression in Wt and ApN^−/−^ microglia after an *in vivo* LPS challenge revealed higher levels of IL-1β, IL-6, and TNFα mRNAs in ApN^−/−^ microglia as compared to Wt microglia (Figure 2E). Altogether, these results indicated that ApN deficiency leads to a greater sensitivity of microglia toward an inflammatory challenge triggered by LPS without significant microglia proliferation.

### ApN limits pro-inflammatory phenotype of brain sorted microglia

**Figure 3:**
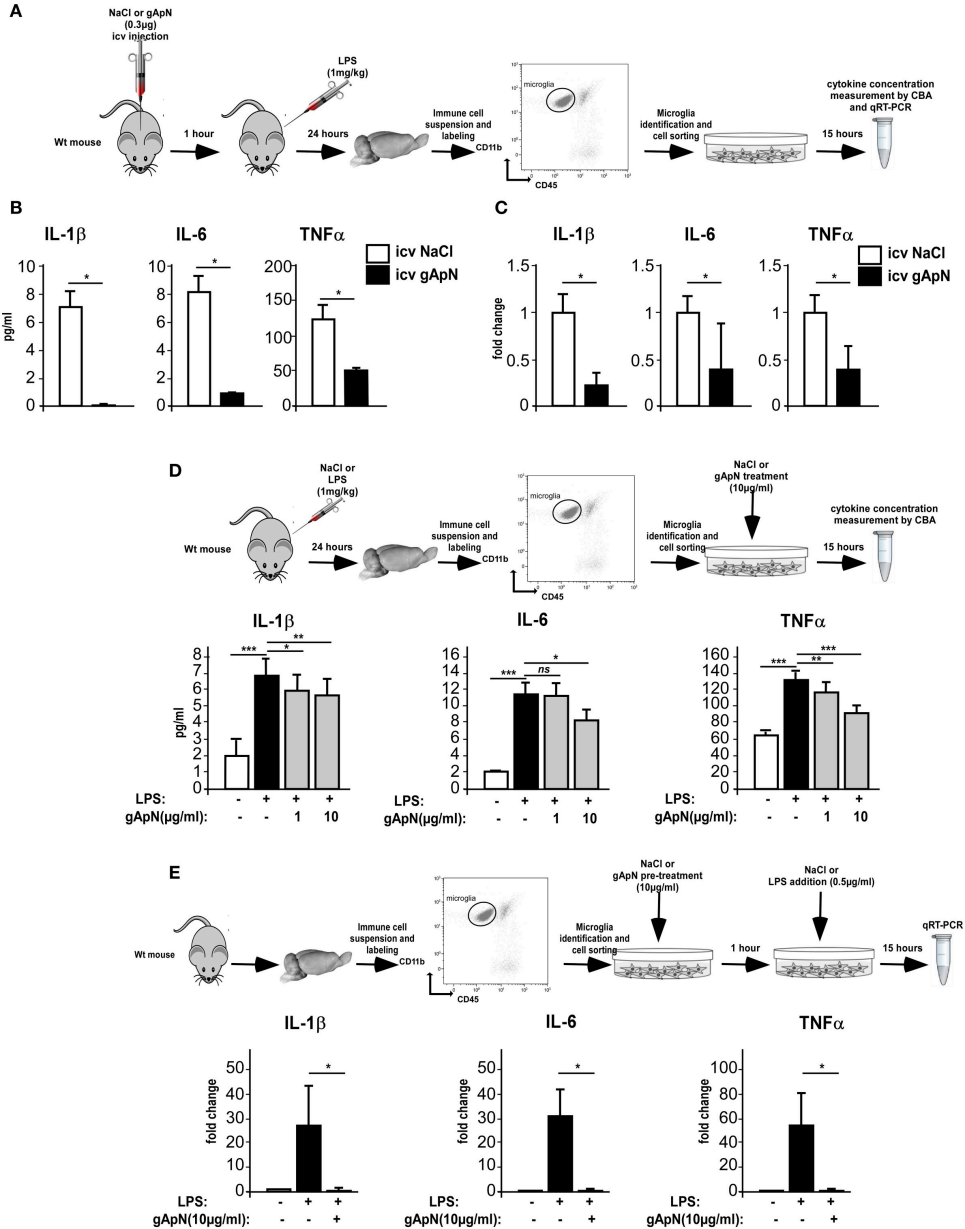
Globular adiponectin limits LPS-induced pro-inflammatory activation of microglia. **(A)** Schematic representation of the protocol. Wt mice first received i.c.v injection of saline or gApN then LPS ip administration. Levels of IL-1β, IL-6, and TNFα proteins **(B)** or mRNAs **(C)** were quantified by CBA or qRT-PCR, respectively, from microglia sorted out from brains of saline (white bars) or gApN (black bars) i.c.v injected mice. *N* = 3 *per* group; Mann & Whitney for comparison between groups, ^*^p < 0.05. **(D)** Twenty-four hours after saline or LPS ip administration, microglia was sorted out from Wt mouse brains and cultured for additional 15 h in the presence of vehicle or gApN in their culture medium. Levels of IL-1β, IL-6, and TNFα proteins were quantified by CBA from microglia sorted out from brains of saline (white bars) or LPS (black bars) treated Wt mice cultured in absence (white and black bars) or presence of 1 or 10 μg/ml gApN (gray bars). *N* = 3 *per* group; Kruskal–Wallis test followed by the Iman-Conover method for multiple comparison between groups was performed, ^*^p < 0.05, ^**^p < 0.01, ^***^p < 0.005; ns, non-significant. **(E)** Microglia was sorted out from Wt mouse brains and cultured for 1 h with saline or gApN as a pre-treatment before addition of LPS. Levels of IL-1β, IL-6, and TNFα mRNAs were quantified by qRT-PCR, from microglia sorted out from brains of saline (white bars) or LPS (black bars) treated microglia cultured in absence (white and black bars) or presence of 10 μg/ml gApN (gray bars). *N* = 3 *per* group; Mann & Whitney for comparison between groups, ^*^p < 0.05.

We next conducted a series of experiments aimed at evaluating the effects of ApN on microglia inflammatory profile. We first examined the *in vivo* effects of ApN on microglia (Figures 3A–C). CBA measurement of cytokines in the secretion media of brain sorted microglia revealed that IL-1β, IL-6, and TNFα levels were significantly lower in the secretion media from microglia sorted out from gApN injected mouse brains as compared to microglia from saline-injected mouse brains (Figure 3B). In agreement with this, qRT-PCR analysis showed that IL-1β, IL-6, and TNFα mRNA levels were significantly lower in microglia sorted out from gApN injected mouse brains as compared to microglia from saline injected mouse brains (Figure 3C). These results suggested that i.c.v injection of gApN significantly, directly or indirectly, prevents the LPS-induced production of pro-inflammatory cytokines by microglia.

To deepen these results, we wondered if gApN could have direct anti-inflammatory effects on microglia. We first tested the ability of gApN to modulate the cytokine production by *in vivo* activated microglia (Figure 3D). Analysis of pro-inflammatory cytokines in the secretion media revealed that microglia sorted out from brains of LPS-treated mice released more IL-1β, IL-6, and TNFα than microglia from saline-treated mice (Figure 3D). *In vitro* treatment of microglia with 1 μg/ml gApN significantly reduced IL-1β and TNFα production, but failed to change IL-6 production (Figure 3D). On the other hand, a higher dose of gApN (10 μg/ml) promoted a greater inhibition of IL-1β and TNFα and also significantly reduced IL-6 release by LPS-activated microglia (Figure 3D). Next, we tested the ability of gApN pre-treatment to block the cytokine production by microglia that has been activated *in vitro* by LPS (Figure 3E). As expected, *in vitro* LPS treatment promoted a strong increase of IL-1β, IL-6, and TNFα mRNAs in microglia that was almost totally abolished by gApN pre-treatment (Figure 3E). Together, these results indicated that gApN has direct anti-inflammatory actions on microglia. Moreover, gApN treatment nonetheless significantly reduced the production of pro-inflammatory cytokines by activated microglia but also efficiently prevented microglia activation when used prophylactically in pre-treatment experiments.

### Globular ApN limits pro-inflammatory activation of different models of microglia

**Figure 4:**
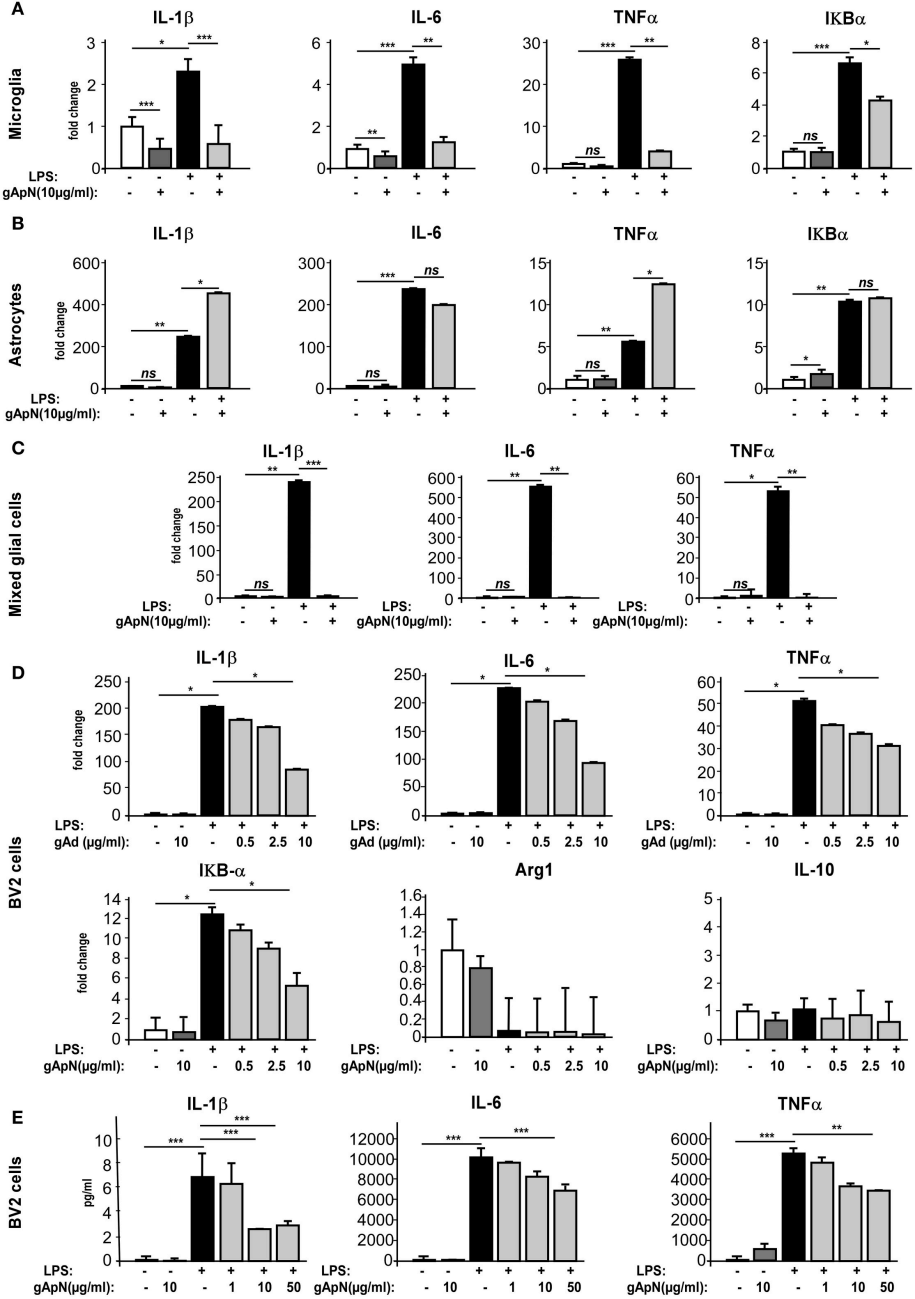
Globular adiponectin limits LPS-induced pro-inflammatory activation of microglia but not astrocytes. **(A–C)** Primary cultures of microglia or astrocytes or both were obtained as described in the Materials and Methods section. Microglia **(A)**, astrocytes **(B)**, mixed glial cells **(C)**, or BV2 cells **(D,E)** were pre-treated with saline (white and black bars) or gApN (gray bars) before treatment with saline (white and dark gray bars) or LPS (black and light gray bars). Levels of IL-1β, IL-6, TNFα, and IκB-α mRNAs **(A–D)** or proteins **(E)** were quantified by qRT-PCR and CBA, respectively. *N* = 3–4 *per* group; Kruskal–Wallis test followed by the Iman-Conover method for multiple comparison between groups was performed, ^*^p < 0.05, ^**^p < 0.01, ^***^p < 0.005; ns, non-significant.

Although, our results indicated that gApN exerted direct anti-inflammatory actions on microglia, we wondered whether gApN could also affect cytokine production by astrocytes, as previously mentioned (Wan et al., 2014). To answer this question, we compared the effects of gApN treatment on primary cultures of microglia, astrocytes, or mixed glial cells obtained from postnatal mouse brains. Microglia or astrocytes were treated with gApN (10 μg/ml) alone, LPS (0.5 μg/ml) alone, or pre-treated with gApN for 1 h before addition of LPS for 15 additional hours (Figure 4). Cells were collected and pro-inflammatory cytokine mRNA levels were analyzed by qRT-PCR. GApN treatment significantly reduced basal levels of IL-1β and IL-6 by microglia but did not modify TNFα mRNA level of unchallenged microglia. Moreover, pre-treatment of microglia with gApN also significantly blocked the increase of IL-1β, IL-6 and TNFα mRNAs elicited by LPS treatment (Figure 4A). In contrast, gApN did not reduce the basal or LPS-induced levels of pro-inflammatory cytokine mRNAs by astrocytes (Figure 4B). Interestingly, gApN treatment on astrocytes rather produced pro-inflammatory effects, since it significantly increased LPS-induced production of IL-1β and TNFα mRNAs (Figure 4B). We also assessed in both cell types the level of expression of IκB-α mRNA. IκB-α is an immediate-early gene induced by LPS challenge and involved in the NF-κB signaling pathway. Here, we confirmed that LPS increased the expression of IκB-α mRNA in both microglia and astrocytes (Figures 4A,B). GApN by itself had no effect on basal expression level of IκB-α mRNA in microglia while it significantly increased it in astrocytes. When used as a pre-treatment, gApN significantly blocked LPS-induced IκB-α mRNA increase in microglia but failed to change it in astrocytes (Figures 4A,B). We also examined gApN effects on primary cultured mixed glial cells containing microglia and astrocytes. Our results showed that gApN (10 μg/ml) successfully limited IL-1β, IL-6, and TNFα mRNA production by mixed glial cells stimulated by LPS (0.5 μg/ml; Figure 4C), suggesting that microglia/astrocytes crosstalk may favor global anti-inflammatory response to gApN. Altogether, these results suggested that gApN exerts different or even opposite effects on microglia and astrocytes but that in a cellular environment containing both microglia and astrocytes (brain, see Figure 1A, or mixed glial cell cultures), gApN treatment elicits rather overall anti-inflammatory effects.

In order to characterize in more detail the anti-inflammatory properties of ApN on microglia, we chose to conduct the following experiments on a more convenient cell model, i.e., the BV2 cell line. Cells were pre-treated for 1 h with saline or gApN (0.5, 2.5, or 10 μg/ml for qRT-PCR experiments, Figure 4D; 1, 10, and 50 μg/ml for CBA assays, Figure 4E) before subsequent LPS addition (0.5 μg/ml), for 15 h. BV2 cells were then collected and cytokine mRNA levels and cytokine secretion were analyzed by qRT-PCR (Figure 4D) and CBA (Figure 4E), respectively. On this cell model, gApN alone had no effect on basal expression levels of any examined mRNA or secreted cytokine. However, we confirmed that a pre-treatment with 10 μg/ml of gApN significantly limited the LPS-induced increase of IL-1β, IL-6, TNFα mRNA expression and protein release (Figures 4D,E). GApN also limited the LPS-induced increase of IκB-α mRNAs (Figure 4D). Our results indicated that gApN dose-dependently decreased the expression of pro-inflammatory markers characteristic of the M1 phenotype (IL-1β, IL-6, TNFα, and IκB-α) of microglia. Therefore, we wondered if gApN could simultaneously promote the expression of anti-inflammatory markers characteristics of the M2 phenotype. We then analyzed Arginase 1 and IL-10 mRNA levels. Our results indicated that neither LPS nor gApN alone or in combination with LPS altered the expression of these markers in the BV2 cell line (Figure 4D). These findings suggested that gApN may block M1 phenotype of microglia rather than promote a M2 phenotype.

### Globular ApN limits nitrosative and oxidative stress in BV2 cells

**Figure 5:**
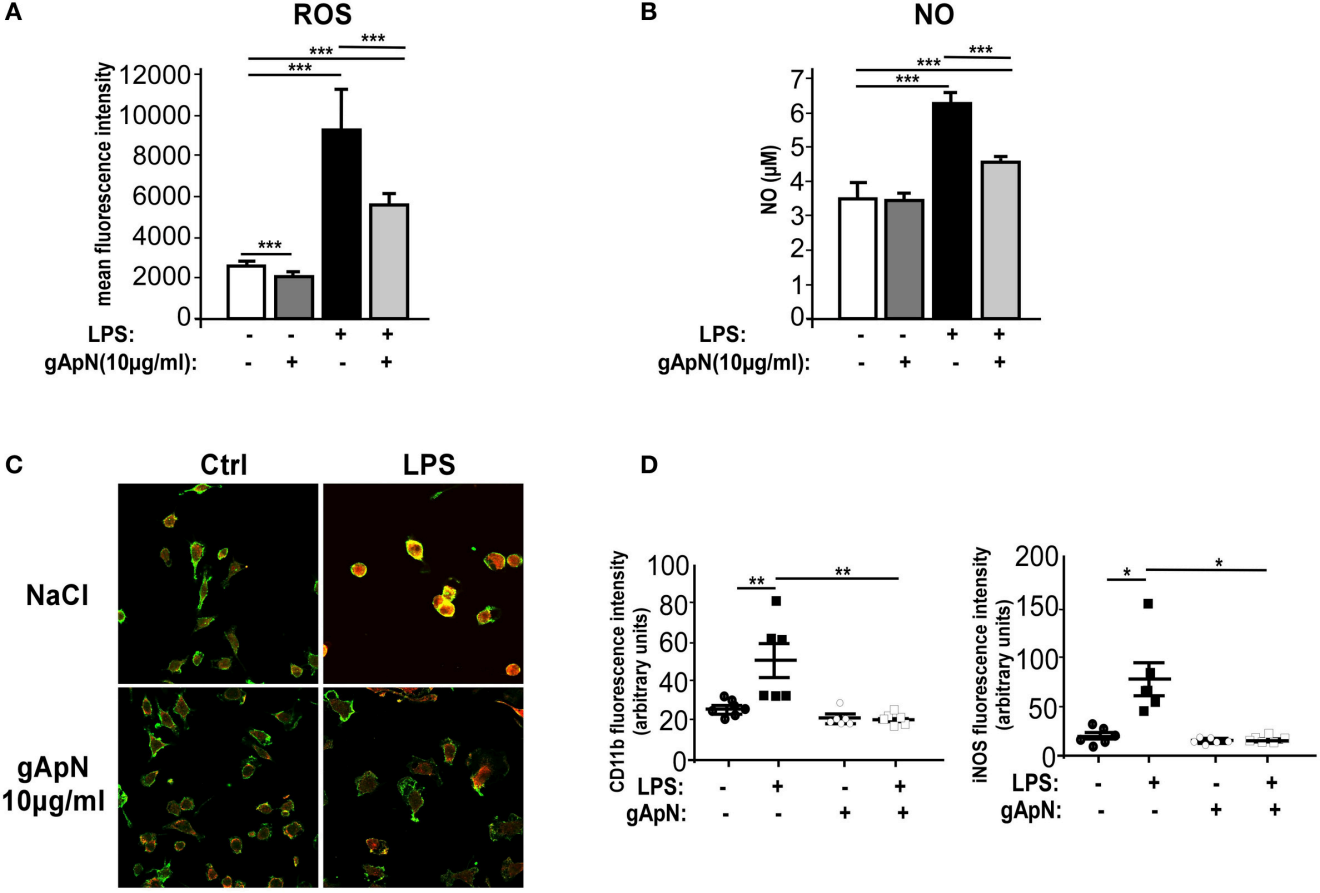
Globular adiponectin limits LPS-induced oxidative and nitrosative stress induction. BV2 cells were pre-treated for 1 h with saline (white and black bars) or gApN (gray bars) before addition of saline (white and dark gray bars) or LPS (black and light gray bars) for 3 **(C,D)** or 15 h **(A,B)**. Reactive oxygen species (ROS) **(A)** and nitric oxide (NO) **(B)** were quantified by flow cytometry and Griess reagent assay, respectively, as described in the Materials and Methods section. *N* = 4–6 per group; Kruskal-Wallis test followed by the Iman-Conover method for multiple comparison between groups was performed, ^***^p < 0.005. **(C)** Representative images of CD11b (green fluorescence) and iNOS (red fluorescence) immunoreactive BV2 cells pre-treated with saline (upper panels) or gApN (lower panels) and treated with saline (left panels) or LPS (right panels). **(D)** Quantification of CD11b and iNOS staining using fluorescence intensity values to quantify their respective expression level. *N* = 6 per group, Kruskal–Wallis test followed by the Iman-Conover method for multiple comparison between groups was performed, ^*^p < 0.05, ^**^p < 0.01.

When activated, microglia produces and releases not only inflammatory cytokines but also reactive oxygen species (ROS) and nitric oxide (NO). To assess whether ApN could regulate ROS and NO induction, BV2 cells were stimulated with LPS only or subsequently to a 1 h pre-treatment with gApN. GApN by itself significantly inhibited the basal level of ROS production in BV2 cells and had no effect on NO basal release. As expected, LPS treatment induced great increases of both ROS and NO production. Both were significantly blocked by gApN pre-treatment (Figures 5A,B). In microglial cells, NO is synthesized from iNOS (Minghetti and Levi, 1998). We thus investigated by immunocytochemistry iNOS immunoreactivity in BV2 cells upon gApN treatment only, LPS only or gApN pre-treatment followed by LPS addition. CD11b labeling was also examined as a control of microglial activation. Images quantification indicated that gApN alone had no effect but reduced both LPS-induced intracellular iNOS and CD11b surface immunoreactivities (Figures 5C,D).

### Globular ApN activates intracellular signaling pathways in BV2 cells

**Figure 6:**
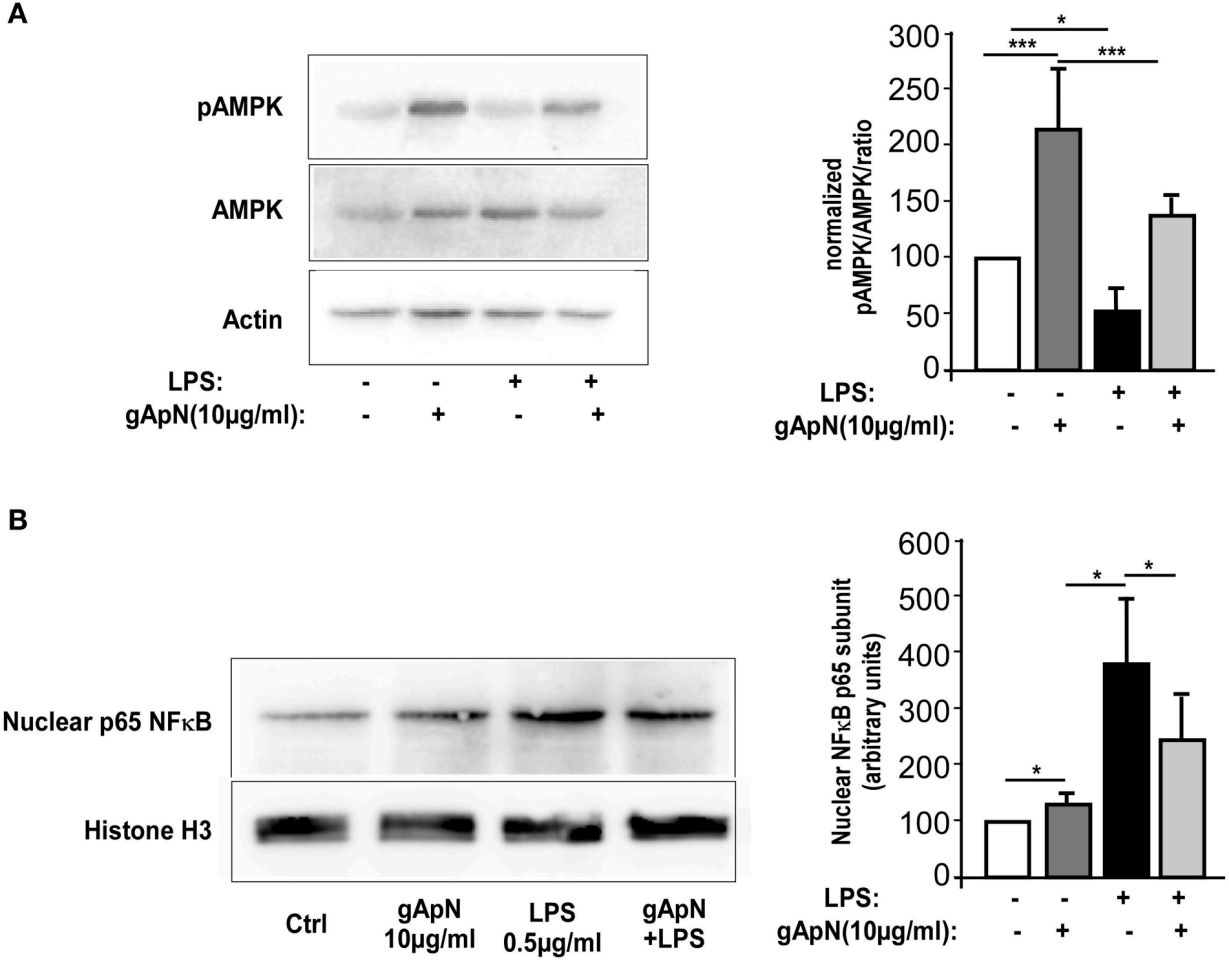
Globular adiponectin activates AMPK phosphorylation and limits NF-κB nuclear translocation. **(A)** Representative western-blot analysis (left panels) of AMPK phosphorylation at Thr172 (pAMPK) in BV2 cells. Right panel shows quantification by densitometry of pAMPK/AMPK ratio of western-blots from 3 independent experiments. Actin immunoreactivity was used for normalization. Mann & Whitney for comparison between groups, ^*^p < 0.05, ^***^p < 0.005. **(B)** Nuclear fractions were obtained from BV2 cells as described in the Materials and Methods section. Left panels show a representative western-blot of the p65 subunit of NF-κB and Histone H3 as a nuclear marker. Right panel shows quantification by densitometry of nuclear p65 immunoreactivity of western-blots from 3 independent experiments. Histone H3 immunoreactivity was used for normalization. Mann & Whitney for comparison between groups, ^*^p < 0.05.

Our next goal was to define downstream pathways that may explain ApN anti-inflammatory, anti-nitrosative, and anti-oxidative actions on microglia cells. In periphery, gApN was suggested to directly regulate glucose metabolism and insulin sensitivity through its action on AMPK (Berg et al., 2001; Tomas et al., 2002; Yamauchi et al., 2002). Moreover, it has been recognized that AMPK signal regulates the inflammatory responses induced by NF-κB (Salminen et al., 2011). However, nothing is known yet on ApN-mediated signaling pathways in microglia. To fill this gap, we analyzed by Western-blotting AMPKα phosphorylation on Thr172 in BV2 cells upon gApN treatment, with or without LPS challenge (Figure 6A). Phosphorylation of AMPKα was significantly enhanced by gApN in basal and LPS-stimulated conditions. In endothelial cells, HMW ApN has been shown to activate AMPK and eNOS, leading to NF-κB dependent signaling pathway regulation (Hattori et al., 2008). We thus analyzed NF-κB activation by studying the nuclear translocation of the p65 subunit in BV2 cells. As expected, nuclear p65 immunoreactivity was increased by LPS treatment. GApN pre-treatment before LPS administration partially blocked the nuclear translocation of p65 (Figure 6B). These results collectively suggest that gApN regulates inflammatory responses by modulating the activity of NF-κB via an AMPK signaling pathway.

### Globular ApN anti-inflammatory actions depend on AdipoR1

**Figure 7:**
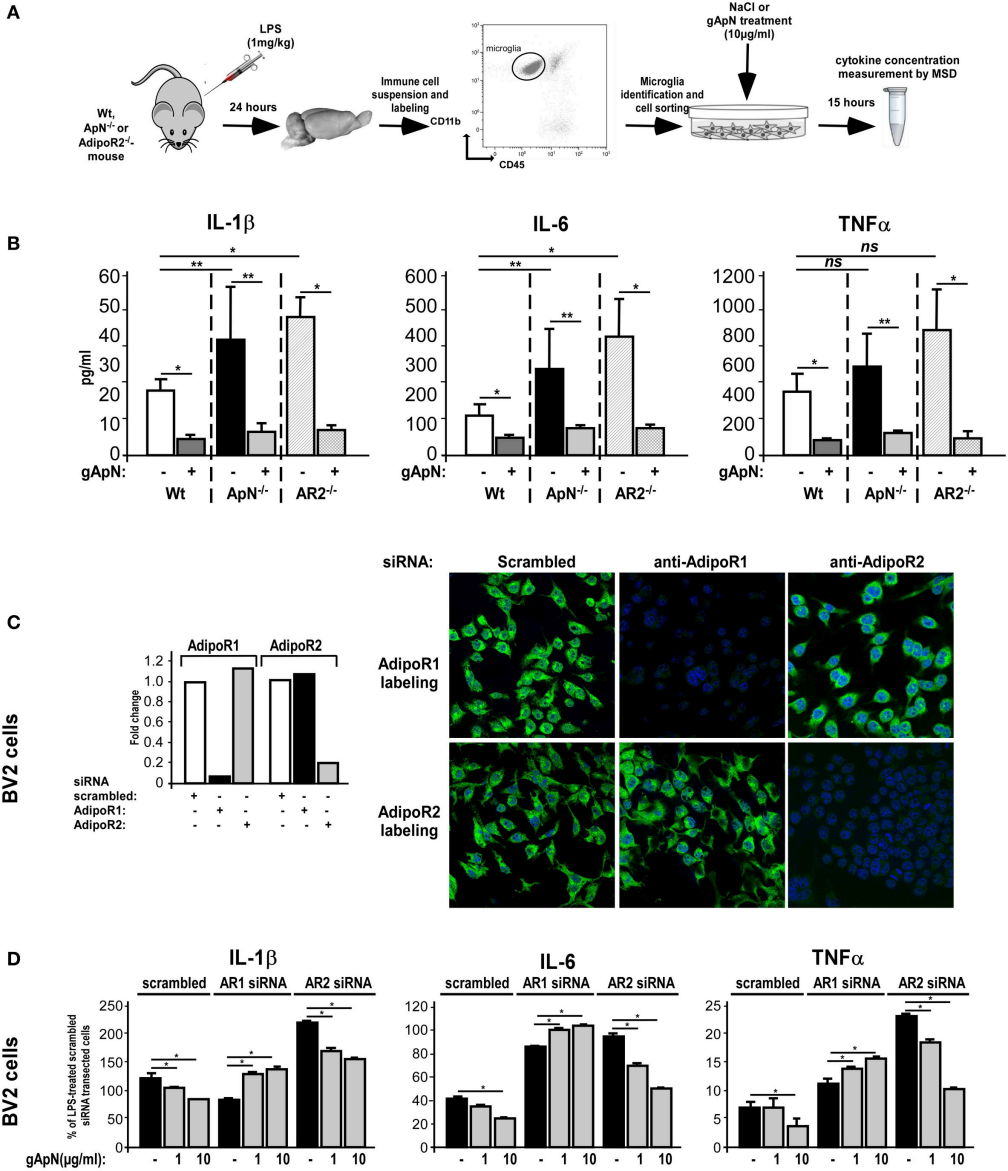
Globular adiponectin anti-inflammatory properties on microglia depend on AdipoR1 expression. **(A)** Schematic representation of the protocol. Wt, ApN^−/−^ and AdipoR2^−/−^ mice were ip administered with LPS and microglia was sorted out from mouse brains and cultured in absence or presence of gApN for 15 h as described in the Materials and Methods section. **(B)** Levels of IL-1β, IL-6, and TNFα proteins released by *in vivo* LPS-activated and *in vitro* saline (white, black, and striped bars) or gApN-treated (dark gray, light gray, and double striped bars) microglia were quantified by MSD. *N* = 4–5 *per* group; Mann & Whitney for comparison between groups, ^*^p < 0.05. **(C)** BV2 cells were transfected with scrambled (white bars), AdipoR1 (black bars), or AdipoR2 (gray bars) specific siRNAs as described in the Materials and Methods section. Forty-eight hours after transfection, AdipoR1 and AdipoR2 expression downregulation was verified by qRT-PCR (histograms) and immunocytochemistry (photographs). Photographs show representative images of BV2 cells transfected with scrambled (left panels), AdipoR1 (middle panels), or AdipoR2 (right panels) specific siRNAs labeled with AdipoR1 (green fluorescence, upper panels) or AdipoR2 antibodies (green fluorescence, lower panels). Nuclei are stained with Hoechst blue fluorescent dye. **(D)** BV2 cells were transfected with scrambled, AdipoR1 or AdipoR2 specific siRNAs, and then pre-treated with saline (black bars) or gApN (gray bars) for 1 h before addition of LPS. Levels of IL-1β, IL-6, and TNFα mRNAs were quantified by qRT-PCR. *N* = 4 *per* group, ns, non-significant; Kruskal–Wallis test followed by the Iman-Conover method for multiple comparison between groups was performed, ^*^p < 0.05; ^**^p < 0.01.

The next step was to study which ApN receptor was responsible for the anti-inflammatory actions of ApN on microglia. Notably, we first noticed by qRT-PCR on microglia sorted out from Wt mouse brains that microglia expressed both AdipoR1 and AdipoR2 mRNAs, at equivalent levels (data not shown). We first subjected Wt, ApN^−/−^ and AdipoR2^−/−^ mice to a single i.p. injection of LPS (1 mg/kg). Twenty-four hours later, microglia was sorted out from mouse brains, plated and treated *in vitro* with saline or gApN (10 μg/ml), for 15 additional hours (Figure 7A). Cytokine level analysis of the secretion media revealed, as previously showed, that LPS-activated microglia from ApN^−/−^ mice produced more IL-1β, IL-6, and TNFα cytokines than that from Wt mice, although differences were not statistically significant for TNFα (Figure 7B). Interestingly, it appeared that LPS-activated microglia from AdipoR2^−/−^ mice also produced significantly more pro-inflammatory cytokines IL-1β and IL-6 than that from Wt mice, suggesting that AdipoR2 deficiency led to a higher susceptibility of microglia toward LPS challenge. However, our findings also revealed that *in vitro* gApN treatment efficiently inhibited IL-1β, IL-6, and TNFα production by Wt, ApN^−/−^, and AdipoR2^−/−^ microglia, showing that AdipoR2 deficiency did not affect gApN anti-inflammatory actions on microglia (Figure 7B).

In periphery, ApN effects have been shown to depend on the molecular structure of the adipokine (globular or full-length) and the expression of the ApN receptor subtypes R1 and/or R2 in target cells (Yamauchi et al., 2002, 2003). However, nothing is known yet about microglial expression of ApN receptors. By qRT-PCR and immunocytochemistry analyzes, we studied AdipoR1 and AdipoR2 expression in BV2 cells. Our results showed that BV2 cells expressed both AdipoR1 and AdipoR2 mRNAs and comparable levels of corresponding proteins (Figure 7C, same results were obtained on brain sorted microglia, data not shown). To deepen AdipoR1 and AdipoR2 involvement in ApN actions on microglia, AdipoR1 or AdipoR2 expression was downregulated with specific siRNAs, cells were pre-treated with saline, 1 or 10 μg/ml of gApN and then treated with LPS (0.5 μg/ml). By qRT-PCR and immunocytochemistry, we showed that siRNA-mediated AdipoR1 or AdipoR2 downregulation led to a very efficient and specific reduction of AdipoR1 or AdipoR2 mRNA and protein expression with only minimal compensatory expression of the other receptor (Figure 7C).

qRT-PCR analyzes of pro-inflammatory cytokine mRNA expression showed that AdipoR1 downregulation blocked the inhibitory actions of gApN on LPS-induced IL-1β, IL-6, and TNFα mRNA upregulation by microglia while AdipoR2 downregulation did not (Figure 7D). It is nonetheless noteworthy to remark that, in agreement with our previous results on microglia sorted out from AdipoR2^−/−^ mouse brains AdipoR2 downregulation in BV2 cells seemed to increase the microglial susceptibility toward LPS challenge, as it expressed more IL-1β, IL-6, and TNFα mRNAs than that transfected with scrambled siRNAs (Figure 7D). Altogether, these findings suggested that AdipoR1 may mediate the anti-inflammatory properties of gApN on microglia, although AdipoR2 may also be involved in global microglial sensitivity toward inflammatory challenges.

### gApN intracellular signaling cascade in microglia depends on AdipoR1 expression

**Figure 8:**
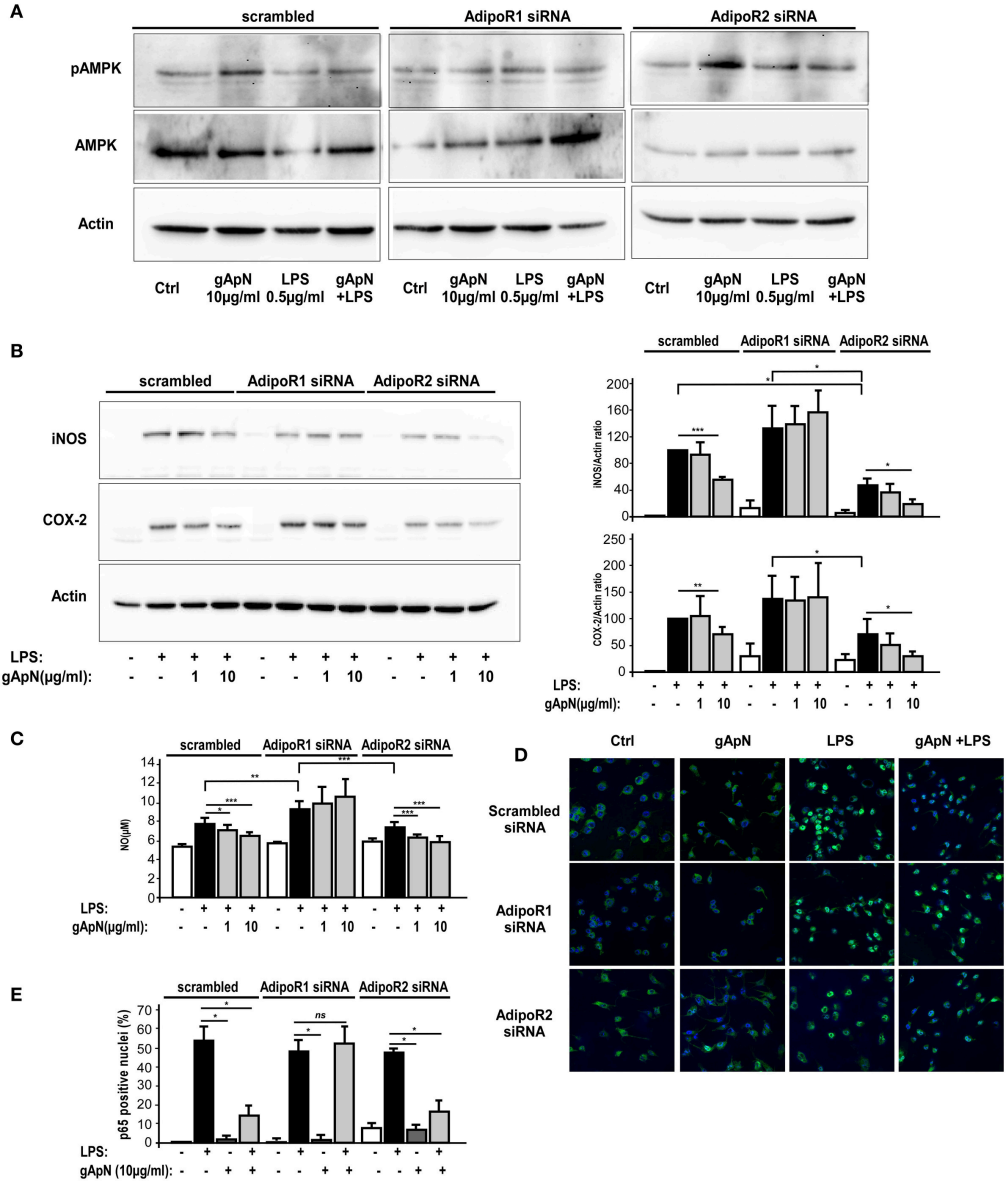
AMPK phosphorylation, anti-oxidative and anti-nitrosative properties of globular adiponectin on microglia depend on AdipoR1 expression. BV2 cells were transfected with scrambled, AdipoR1 or AdipoR2 specific siRNAs as described in the Materials and Methods section. Forty-eight hours after transfection, cells were pre-treated with saline or gApN 1 h before addition of LPS for 15 h. **(A,B)** Representative western-blot analysis of LPS, gApN, and gApN+LPS treatment on AMPK phosphorylation on Thr172 **(A)**, iNOS (**B**, upper panel) and COX-2 (**B**, lower panel) immunoreactivities in BV2 cells transfected with scrambled (left panels), AdipoR1 (middle panels), or AdipoR2 (right panels) specific siRNAs. Actin immunoreactivity is used as a loading control. **(B)** Histograms show western-blot quantification of iNOS (upper histograms) and COX-2 (lower histograms) immunoreactivities in siRNA transfected BV2 cells pre-treated with saline (white and black bars) or gApN (gray bars) before addition of saline (white bars) or LPS (black and gray bars). **(C)** Nitric oxide (NO) release was quantified in medium of siRNA transfected BV2 cells pre-treated with saline (white and black bars) or gApN (gray bars) before addition of saline (white bars) or LPS (black and gray bars). *N* = 4 per group; Kruskal–Wallis test followed by the Iman-Conover method for multiple comparison between groups was performed, ^*^p < 0.05, ^**^p < 0.01, ^***^p < 0.005. **(D)** Representative photomicrographs of p65 NF-κB subunit immunoreactivity (green fluorescence) in BV2 transfected with scrambled (upper panels), AdipoR1 (middle panels), or AdipoR2 (lower panels) siRNAs pre-treated with saline or gApN before addition of LPS. Nuclei are stained with Hoechst blue fluorescent dye. **(E)** Quantification of p65 positive nuclei expressed as percentage of total nuclei in siRNA transfected BV2 cells pre-treated with saline (white and black bars) or gApN (gray bars) before addition of saline (white bars) or LPS (black and gray bars). *N* = 4, Mann & Whitney for comparison between groups, ^*^p < 0.05, ns, non-significant.

We next studied AdipoR1 and AdipoR2 involvements in gApN-dependent AMPKα phosphorylation and downstream intracellular molecular targets. By Western-blotting, we showed that AdipoR1 downregulation totally blocked the gApN-induced phosphorylation of AMPKα, while AdipoR2 downregulation did not change it (Figure 8A).

NF-κB signaling pathways are activated in response to extracellular stimuli, including LPS, leading to the induction of various genes, including iNOS and COX-2, in microglia (Dang et al., 2014). Thus, we studied iNOS and COX2 immunoreactivities in BV2 cells transfected with scrambled, AdipoR1 or AdipoR2 siRNAs, and treated with LPS only or pre-treated with gApN (1 or 10 μg/ml) before addition of LPS (0.5 μg/ml) for 15 h. Results revealed that, as expected, LPS induced a great increase of both iNOS and COX2 expression in scrambled siRNA-transfected BV2 cells that was significantly reduced by the highest dose (10 μg/ml) of gApN (Figure 8B). GApN inhibitory effects were totally abolished in cells where AdipoR1 was downregulated whereas they remained significant in cells transfected with siRNA-targeted AdipoR2 (Figure 8B). In agreement with these results, LPS-stimulated NO release was reduced by gApN in cells transfected with scrambled or AdipoR2-targeted siRNAs while it was not modified in cells transfected with AdipoR1-targeted siRNAs (Figure 8C).

**Figure 9:**
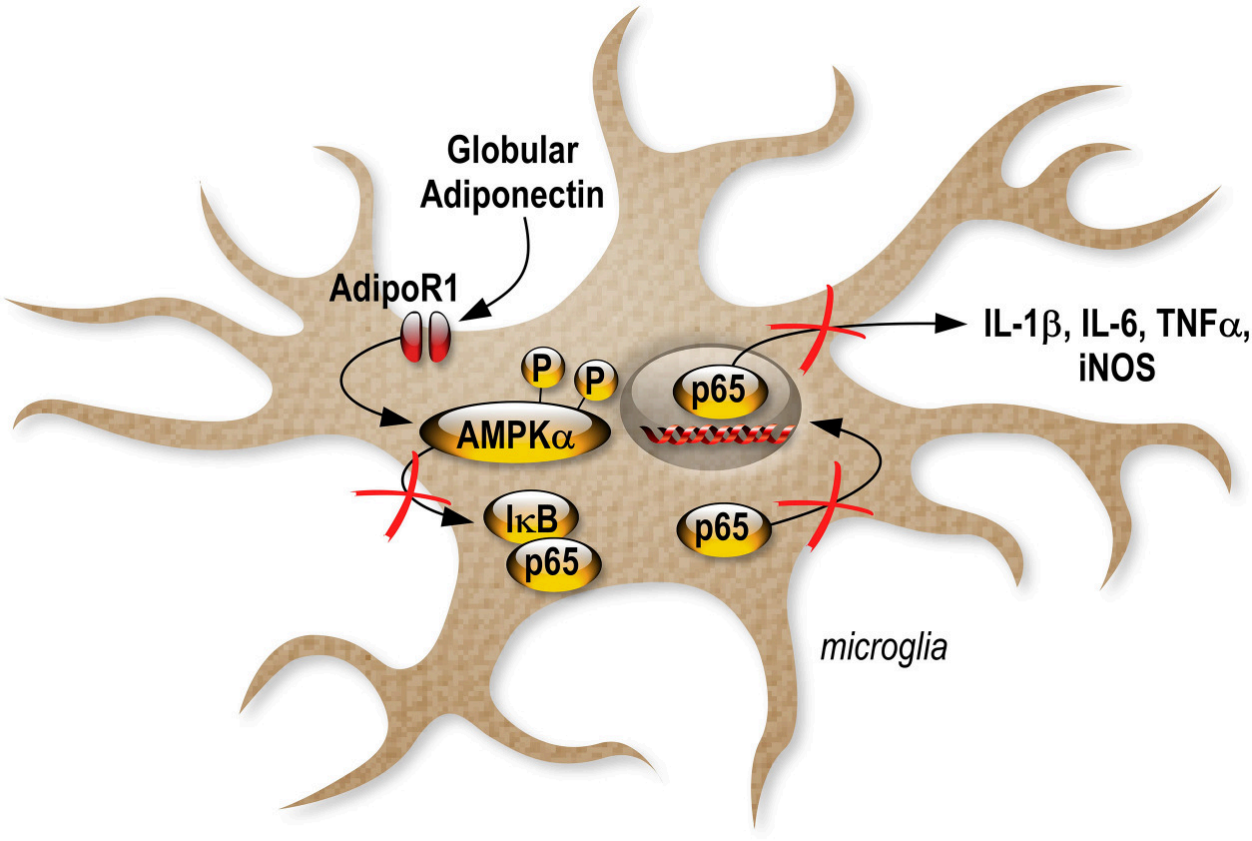
Schematic illustration revealing the anti-inflammatory effects of gApN on microglia.

We also investigated gApN effect on p65 NF-κB nuclear translocation by immunocytochemistry in siRNA-transfected cells. As previously showed by Western-blotting of nuclear fractions (Figure 6B), analysis of immunocytochemistry images revealed that percentage of p65 positive nuclei was increased by LPS treatment in scrambled-, AdipoR1 siRNA-, AdipoR2 siRNA- transfected cells. GApN pre-treatment before LPS administration partially blocked the percentage of p65 positive nuclei in scrambled- and AdipoR2 siRNA-transfected cells but not in AdipoR1 siRNA-transfected cells (Figures 8D,E). Globally, our findings indicated that gApN anti-inflammatory and anti-oxidant actions on microglia are mediated through an AdipoR1/AMPK/NF-κB dependent signaling pathway (Figure 9).

### AdipoRon treatment fails to exert anti-inflammatory effects on mouse brain and BV2 cells

Next, we tested whether an AdipoR agonist, AdipoRon, could also exert anti-inflammatory effects on brain and microglia. Wt mice were chronically treated with vehicle or 1 mg/kg AdipoRon for 15 consecutive days and then administered with a single i.p. injection of saline or LPS (Table 1). As expected, LPS injection induced a significant increase in IL-1β, IL-6, and TNFα protein levels in prefrontal cortex, hippocampus and hypothalamus but AdipoRon chronic pre-treatment failed to prevent these pro-inflammatory effects (Table 1), although it crosses blood-brain barrier (unpublished observations).

**Table 1:** Effect of AdipoRon treatment on cytokine concentration in brain areas of Ctrl or LPS-injected mice.

**pg/mg of protein**	**IL-1β**				**IL-6**				**TNFα**			
AdipoRon	–	+	–	+	–	+	–	+	–	+	–	+
LPS	–	–	–	–	+	+	+	+	–	–	+	+
Prefrontal cortex	0.4 ±0.04	0.4 ± 0.06	1.6 ± 0.2	2.1 ± 0.1	8.8 ± 2.1	8.1 ±1.0	1.4 ± 0.2	1.55 ± 0.2	0.05 ± 0.01	0.07 ± 0.03	0.4 ±0.05	0.5 ± 0.05
Hypothalamus	0.7 ±0.2	0.7 ± 0.3	2.6 ± 0.7	2.9 ± 0.8	11.8 ± 2.2	14.3 ±3.5	1.5 ± 0.2	1.8 ± 0.5	0.16 ± 0.09	0.3 ± 0.1	0.47 ±0.1	0.65 ± 0.08
Hippocampus	0.3 ±0.03	0.3 ± 0.04	1.7 ± 0.3	2.4 ± 0.2	9.2 ± 1.5	9.9 ±1.7	1.2 ± 0.1	1.9 ± 0.4	0.03 ± 0.01	0.03 ± 0.02	0.55 ±0.08	0.6 ± 0.04

Wt mice (10 weeks old) were daily i.p. injected with vehicle (2.5% DMSO solution) or AdipoRon (1 mg/kg in 2.5% DMSO solution) during 15 consecutive days. On day 15, mice received a single i.p. injection of saline or LPS (0.8mg/kg). Twenty-four hours later, mice received a last injection of AdipoRon and were sacrificed. Brains were dissected and pro-inflammatory cytokines were measured by MSD in different brain regions. Data represent means ± sem, n = 5 mice for LPS treated group without AdipoRon and n = 8 for LPS + AdipoRon treated group. A two-way ANOVA comparing the effect of AdipoRon in untreated and treated LPS groups showed no significant interaction while the effect of LPS was significant in all considered brain regions

**Figure 10:**
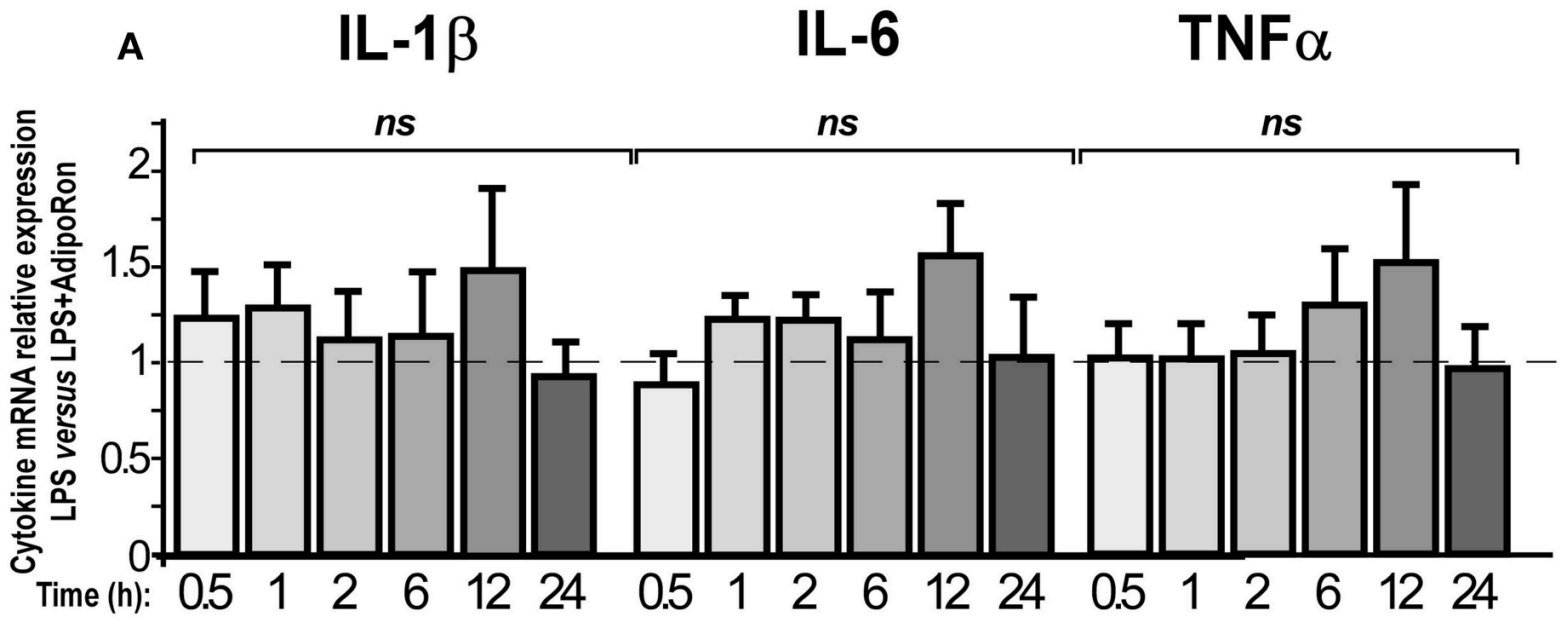
AdipoRon, a small AdipoR agonist, doesn't exert anti-inflammatory effects on BV2 cells. BV2 cells were pre-treated with vehicle or AdipoRon (10^−6^ M) before treatment with LPS (0.5 μg/ml) for the indicated period of time. Levels of IL-1β, IL-6, TNFα mRNAs were quantified by qRT-PCR. *N* = 5–7, two-way analysis of variance (ANOVA) was carried out, ns, non-significant.

We also examined whether AdipoRon could exert direct anti-inflammatory actions on BV2 cells (Figure 10). AdipoRon pre-treatment (10^−6^ M) did not statistically modify IL-1β, IL-6, and TNFα mRNA expression levels in LPS-treated BV2 cells as compared to vehicle pre-treatment, whatever the incubation period considered (Figure 10). Dose-response experiments were also performed but none of the concentrations tested limited the LPS-induced pro-inflammatory cytokines mRNA levels increase (data not shown).

## Discussion

In the present study, we show that ApN deficiency results in higher susceptibility of the brain toward an inflammatory challenge elicited by LPS. This exacerbated sensitivity is due to microglia increased responsiveness. Also, we have identified the presence of both ApN receptors (AdipoR1 and AdipoR2) on microglia. On different *in vivo* and cultured models of microglia (brain-sorted *ex vivo* cultured microglia, primary cultured microglia, and BV2 cell line), we demonstrate that gApN has direct anti-inflammatory actions on microglia that limit LPS-induced IL-1β, IL-6, and TNFα release. GApN properties are effective preventively, by blocking the activation of microglia by LPS, or *a posteriori*, by limiting the production of proinflammatory cytokines by LPS-activated microglia. According to our results, gApN would limit the M1 activation state of microglia without promoting the expression of M2 markers, such as Arginase 1 or IL-10. These effects on microglia differ from those describing that, in periphery, ApN promotes macrophage polarization toward an anti-inflammatory M2 phenotype both *in vivo* and in cultured macrophages (Ohashi et al., 2010; Mandal et al., 2011). However, it has been shown that the M2-promoting effects of ApN would be mediated in an AdipoR2-dependent manner (Mandal et al., 2011). Here, we show that the anti-inflammatory effects of gApN on microglia are mediated by AdipoR1, which could explain the difference.

Anti-inflammatory properties of gApN cannot be generalized to all brain cell types. Indeed, in a previous study, Wan et al. showed that gApN exerts rather pro-inflammatory effects on human astrocytic cells by inducing IL-6 and MCP-1 secretion and gene expression of IL-6, MCP-1, IL-1β, and IL-8 (Wan et al., 2014). In agreement with this, our results show that gApN increases LPS-induced IL-1β and TNFα gene expression by mouse astrocytes. Noteworthy, gApN still has overall anti-inflammatory effects on mixed primary cultures of microglia and astrocytes, indicating that the anti-inflammatory actions of gApN on microglia are predominant compared to the pro-inflammatory ones on astrocytes. This is also true when examining the *in vivo* global effects of gApN on diverse brain regions such as hippocampus, hypothalamus or amygdala (Figure 1). The role of microglia and astrocyte crosstalk in neuroinflammation is poorly understood. However, we can speculate that when both are present and interact, microglia may be able to modulate astrocytic activation. This hypothesis is supported by a recent study showing that metal manganese induces an inflammatory phenotype in microglia that is essential for the subsequent activation of astrocytes (Kirkley et al., 2017). Moreover, these opposite effects of gApN on astrocytes and microglia comfort us to exclude the possibility that the anti-inflammatory actions of gApN may result from its ability to bind LPS, as suggested previously (Peake et al., 2006). Indeed, Peake et al. demonstrated that both recombinant and native ApN directly bind LPS and may act as a scavenging anti-inflammatory agent (Peake et al., 2006). In such a case, anti-inflammatory effects of gApN treatment would be expected on both astrocytes and microglia.

AMPKα is a signaling kinase involved in a critical energy-sensing pathway with important functions in stimulating glucose uptake. Our present study demonstrates that AMPKα phosphorylation is down-regulated by LPS and up-regulated by gApN in BV2 microglia cells. It is known that pharmacological activation of AMPKα by 5-aminoimidazole-4-carboxamide-1-β-d-ribofuranoside (AICAR) inhibits iNOS induction in macrophages and microglia exposed to LPS (Kuo et al., 2008). Gene-silencing experiments confirmed that AMPK-activating agents blunt iNOS-mediated NO production, at least in part, via activation of AMPKα (Pilon et al., 2004). Therefore, although we did not formally demonstrate it in this present study, we can hypothesize that gApN regulates iNOS expression and NO production through AMPKα activation in microglia.

Reactive oxygen species (ROS) are crucial regulators of microglial inflammatory function since they act as secondary messengers capable of modifying pro-inflammatory gene expression through effects on kinase cascades and activation of transcription factors, including MAPK and NF-κB (Torres and Forman, 2003; Pawate et al., 2004; Kim et al., 2008). One potential source of ROS production is NADPH oxidase in the plasma membrane and mitochondria (Collins et al., 2012). ROS generated from NADPH oxidase are involved in the signaling events leading to microglia activation (Cheret et al., 2008). Conversely, inhibition of NADPH oxidase prevents NF-κB-dependent iNOS expression and NO production in LPS-stimulated macrophages (Kim et al., 2009). Therefore, it is likely that neutralization of mitochondrial ROS can alleviate inflammation (Voloboueva et al., 2013). Here, we show that gApN limits LPS-induced ROS production and NF-κB p65 nuclear translocation. This would lead to a reduction of oxidative stress-related iNOS and COX-2 enzymes induction, thereby limiting NO and subsequent pro-inflammatory cytokine release production in BV2 cells. This is consistent with the previous observation made in vascular endothelial cells showing that HMW ApN activates AMPK and suppresses cytokine-induced NF-κB activation (Hattori et al., 2008). Interestingly, if the inhibitory actions of gApN on oxidative stress have already been described in different cell models such as human hepatic cells (Shrestha and Park, 2016) or macrophages (Kim et al., 2014), its effects on nitrosative stress are more controversial and seem to be highly dependent on the cell type. For example, gApN has been shown to induce NO production in vascular endothelial cells (Cheng et al., 2007; Dong et al., 2015). However, in advential fibroblasts, it reduces LPS-induced NO production by inhibiting AdipoR1/AMPK/iNOS pathway (Cai et al., 2010), which is a signaling pathway similar to the one we describe in the present manuscript. Indeed, our results show that the effects of gApN on microglia may be mediated by AdipoR1, since its anti-inflammatory and anti-nitrosative properties are abrogated by siRNA-targeted AdipoR1 downregulation in BV2 cells and that AdipoR2^−/−^ brain-sorted microglia are still sensitive to gApN anti-inflammatory effects. Nevertheless, we cannot formally exclude the involvement of AdipoR2 in the effects of ApN on microglia. First, our results suggest that siRNA-targeted AdipoR2 downregulation modifies microglia intrinsic characteristics. Indeed, it increases the LPS-induced production of pro-inflammatory cytokines IL-1β, IL-6, and TNFα and decreases LPS-induced iNOS expression. This indicates that AdipoR2 expression level somehow regulates microglia responsiveness toward an inflammatory challenge but does not mediate gApN effects on microglia. It is noteworthy that most of our experiments were conducted to study the effect of the globular form of ApN. GApN has been proposed to be generated from full-length by proteolytic cleavage by leukocyte elastase, secreted from activated monocytes and/or neutrophils (Waki et al., 2005). The AdipoRs display differential binding affinities for the various ApN multimers. While AdipoR1 binds to gApN with high affinity, AdipoR2 has an intermediate binding affinity for both gApN and full-length ApN (FL-ApN) (Yamauchi et al., 2003). The biological activities of FL-ApN and gApN do not necessarily overlap since it has been shown, for example, that gApN but not FL-ApN induces increased procoagulability in human endothelial cells (Bobbert et al., 2008). Although we did not perform all the experiments presented here with FL-ApN to compare its actions with those of gApN, we obtained results showing that FL-ApN also significantly prevented LPS-induced microglia activation when injected intracerebroventricularly (Figures S1A,B). In scrambled siRNA-transfected BV2 cells, FL-ApN also limited LPS-induced increases of IL-1β, IL-6, and TNFα mRNAs. Interestingly, this effect was still present in cells transfected with siRNA-targeted AdipoR2, whereas it was abolished in cells transfected with siRNA-targeted AdipoR1 (Figure S1C). This suggests that anti-inflammatory actions of ApN on microglia are not limited to its globular form and that FL-ApN effects on microglia are probably also dependent on AdipoR1 expression. Intriguingly, our results show that AdipoRon, an AdipoR agonist that binds to both AdipoR1 and AdipoR2 with comparable high affinities (Okada-Iwabu et al., 2013), neither prevents LPS-induced neuroinflammation when preventively chronically administered to mice nor blocks LPS-induced BV2 pro-inflammatory activation. One possible explanation might lie in the fact that in cells expressing comparable levels of both AdipoR1 and AdipoR2 (such as microglia and BV2 cells), agonist binding-induced intracellular signaling pathways may lead to opposite effects which mask the AdipoR1-dependent anti-inflammatory effects. Additional experiments would be necessary to address this question.

In previous studies we have shown that favorable living conditions such as those mimicked by EE could favor the passage of the LMW forms of ApN from the blood to the CSF where they influence microglia activation state thereby limiting neuroinflammation (Chabry et al., 2015). We have shown that ApN-mediated decrease in neuroinflammation efficiently limited anxio-depressive-like behavior in mice (Nicolas et al., 2015). It has been previously shown that ApN has beneficial effects on brain ischemic injury through an endothelial NO synthase-dependent mechanism. Indeed, ApN-KO mice have more serious damage than WT mice after ischemia-reperfusion as illustrated by enlarged brain infarction and increased neurological deficits. Conversely, administration of full-length ApN through adenovirus significantly reduced cerebral infarct size in WT and ApN-KO mice (Nishimura et al., 2008). More generally, anti-inflammatory actions of ApN in CNS could constitute a means of combating various mental disorders and brain diseases associated with deleterious chronic inflammation or exacerbated inflammatory response, such as depression, anxiety, schizophrenia, stroke, multiple sclerosis, Parkinson's disease, and Alzheimer's disease.

## Conclusions

Together, our results provide the first direct evidence demonstrating that gApN acts against neuroinflammation by an AdipoR1-AMPK-NF-κB pathway, as illustrated by the inhibition of LPS-mediated pro-inflammatory cytokine, NO and ROS production in microglia (Figure 9).

## Availability of data and materials

The datasets used and/or analyzed during the current study are available from the corresponding author on reasonable request.

## Author contributions

SN, HZ, and AP-P performed experiments. JCa carried out the flow cytometry, cell sorting, and cytometric bead array experiments. AP-P coordinated the study, analyzed the data, and drafted the manuscript with the assistance of JCh and AG. CH provided helpful advice and fruitful discussion. All authors have read and approved the final version of the manuscript.

### Conflict of interest statement

The authors declare that the research was conducted in the absence of any commercial or financial relationships that could be construed as a potential conflict of interest.

## Supplementary material

Figure S1 Full-length adiponectin limits LPS-induced pro-inflammatory activation of microglia. **(A)** Schematic representation of the protocol. Wt mice first received i.c.v injection of saline or FL-ApN then LPS ip administration. **(B)** Levels of IL-1β, IL-6, and TNFα mRNAs were quantified qRT-PCR, from microglia sorted from brains of saline (white bars) or FL-ApN (black bars) i.c.v injected mice. *N* = 3 *per* group; Mann & Whitney for comparison between groups, ^*^*p* < 0.05. **(C)** BV2 cells were transfected with scrambled, AdipoR1 or AdipoR2 specific siRNAs as described in the Materials and Methods section. Forty-eight hours after transfection, cells were pre-treated with saline or FL-ApN 1 h before addition of LPS for 15 additional hours. Levels of IL-1β, IL-6, and TNFα mRNAs were quantified by qRT-PCR. *N* = 3 *per* group, ns, Mann & Whitney for comparison between groups, ^*^*p* < 0.05.

## Abbreviations

ApN: adiponectin
gApN: globular adiponectin
FL-ApN: Full-length adiponectin
LPS: lipopolysaccharide
IL: interleukin
TNF: tumor necrosis factor
AdipoR1: adiponectin receptor 1
AdipoR2: adiponectin receptor 2
MDD: major depressive disorders
NF-κB: nuclear factor-kappa B
CNS: central nervous system
EE: enriched environment
CSF: cerebrospinal fluid
CBA: cytometric bead array™
MSD: Meso Scale Discovery
ip: intraperitoneal
i.c.v.: intracerebroventricular
FSC: forward scatter
SSC: side scatter
ROS: reactive oxygen species
NO: nitric oxide
CD: cluster of differentiation
AMPK: AMP-activated protein kinase
MAPK: Mitogen-activated protein kinase
iNOS: inducible nitric oxide synthase
COX-2: cyclooxygenase-2.
