# Supplementary figures and images for: Globular Adiponectin Limits Microglia Pro-Inflammatory Phenotype through an AdipoR1/NF-κB Signaling Pathway
Source: Front Cell Neurosci. 2017 Nov 14;11:352. doi: 10.3389/fncel.2017.00352 (PMC5694456; doi:10.3389/fncel.2017.00352)

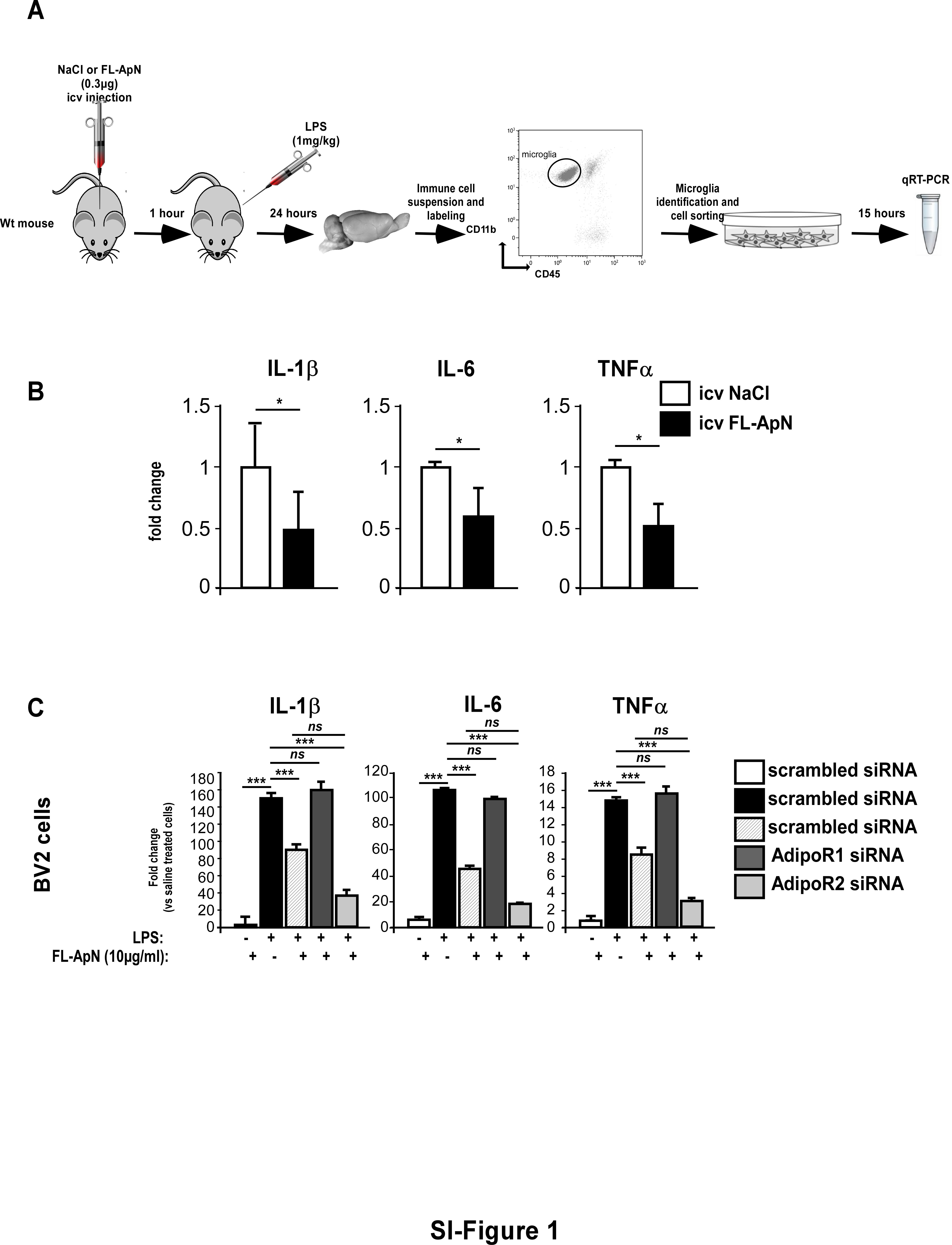

Supplement: Figure S1 — Full-length adiponectin limits LPS-induced pro-inflammatory activation of microglia. (A) Schematic representation of the protocol. Wt mice first received i.c.v injection of saline or FL-ApN then LPS ip administration. (B) Levels of IL-1β, IL-6, and TNFα mRNAs were quantified qRT-PCR, from microglia sorted from brains of saline (white bars) or FL-ApN (black bars) i.c.v injected mice. N = 3 per group; Mann & Whitney for comparison between groups, *p < 0.05. (C) BV2 cells were transfected with scrambled, AdipoR1 or AdipoR2 specific siRNAs as described in the Materials and Methods section. Forty-eight hours after transfection, cells were pre-treated with saline or FL-ApN 1 h before addition of LPS for 15 additional hours. Levels of IL-1β, IL-6, and TNFα mRNAs were quantified by qRT-PCR. N = 3 per group, ns, Mann & Whitney for comparison between groups, *p < 0.05. [file Image1.jpg]
